# Hyaluronic acid-coated capecitabine nanostructures for CD44 receptor-mediated targeting in breast cancer therapy†

**DOI:** 10.1039/d5ra01275a

**Published:** 2025-04-22

**Authors:** Sruthi Laakshmi Mugundhan, Mothilal Mohan

**Affiliations:** a Department of Pharmaceutics, SRM College of Pharmacy, SRM Institute of Science and Technology Kattankulathur-603203 Chengalpattu Tamil Nadu India mothipharma78@gmail.com

## Abstract

Hyaluronic acid-coated capecitabine-loaded nanomicelles (HA-CAP-M) are synthesized to overcome the challenges associated with capecitabine (CAP) conventional delivery such as low permeability and systemic toxicity. Nanomicelles containing saponin, glycerol, and vitamin-E TPGS formulation of capecitabine were further encapsulated with hyaluronic acid (HA) for CD44 receptor-mediated targeting. Optimization of the formulation was carried out using a Box–Behnken design resulting in 17.8 nm particle size, 89.3% entrapment efficiency and a biphasic drug release profile. Characterization studies validated stability, spherical structure, and desirable encapsulation characteristics of the nanomicelles. Lowered critical micelle concentration (CMC) and acceptable drug release kinetics revealed improved thermodynamic stability and controlled drug release, as predicted by the Hixson–Crowell model. HA-CAP-M showed much higher permeability and cytotoxicity than the free CAP, with an IC_50_ of 2.964 μg mL^−1^ in *in vitro* experiments. AO/PI staining also demonstrated dose-dependent apoptosis in MCF-7 breast cancer cells and validated the highly effective active targeting of HA. In addition, the formulation demonstrated good stability during storage and dilution conditions, confirming its stability as a drug delivery platform. In conclusion, HA-functionalized nanomicelles provide a biocompatible and efficient system for the targeted breast cancer therapy, enhancing the therapeutic efficacy of capecitabine.

## Abstract 2

Hyaluronic acid-coated capecitabine nanomicelles enhance drug delivery by improving permeability, targeting CD44 receptors, and inducing dose-dependent apoptosis in breast cancer cells, offering a stable and efficient therapy.

## Graphical abstract

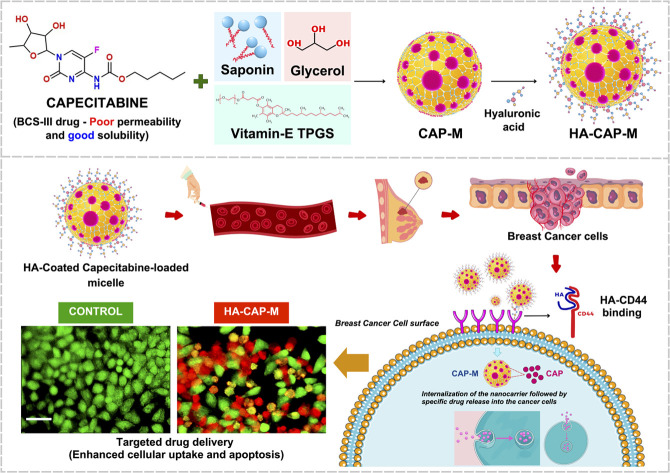

## Introduction

Cancer is a condition characterised by unregulated/uncontrolled proliferation of cells with the failure to undergo programmed cell death, resulting in the destruction of adjacent tissues.^1^ Breast cancer (BC) was the most common cancer in women globally in 2022, as per the *Global Cancer Statistics Report by WHO*, 2024.^2^ Female BC ranked second in the most common cancer types among 36 cancers in 186 countries, with about 2.3 million cases covering 11.6% of the total with 670 000 fatalities globally in the same year.^3^ Incidences of breast cancer rose by 130% from 2008 to 2020, increasing from 1.38 million new cases to 1.67 million in 2012, 2.1 million in 2018, and 2.3 million in 2020.^4^

Despite advancements in imaging technology and therapeutic methods, BC continues to result in numerous fatalities.^5^ BC impacts both the quality of life and the survival probability of patients. BC patients have access to various treatment modalities, including medication, chemotherapy, and surgical interventions, based on the specific type and location of the cancer. Conventional drug therapies exhibit limitations due to the inefficiencies in anti-cancer drug delivery. Nanomedicines utilising nanocarriers present promising therapeutic agents for breast cancer, addressing the limitations of potential drugs.^6,7^

Nanomicelles are small colloidal structures that consist of molecules with two distinct regions: a core and a shell, which possess different affinities for water.^8^ Poorly water-soluble drugs are encapsulated in the hydrophobic core of nanomicelles, protected by a hydrophilic corona that stabilizes the micelles and minimizes recognition by the reticuloendothelial system (RES) *in vivo*.^9^ This unique structure enables nanomicelles to enhance drug solubility and bioavailability, protect drugs from degradation, and facilitate transport across biological barriers.^10^ Their ability to accumulate in diseased tissues *via* the enhanced permeability and retention (EPR) effect allows targeted drug delivery with reduced systemic toxicity, making them valuable for therapeutic and diagnostic applications.^11^

Nanomicelles offer thermodynamic and kinetic stability due to the intra-micellar polymer chain entanglement.^12^ Thermodynamic stability arises when nanomicelles exceed their critical micelle concentration (CMC), typically low (10^−6^ to 10^−7^ M), ensuring stability even after dilution. Kinetic stability allows nanomicelles to retain drugs under unpredictable release conditions, with slow disintegration below the CMC, preserving drug content until delivery to the target site, thus enhancing bioavailability.^13^

Nanomicelles improve treatment efficacy by enhancing drug selectivity and specificity, minimizing adverse effects, and reducing exposure to non-target tissues.^14,15^ Their enhanced permeability and retention (EPR) effect increases drug concentration in tumours, overcoming drug resistance.

Compared to liposomes, nanoparticles, and nanotubes, nanomicelles exhibit superior performance in cancer therapy due to their nanoscale size, high drug-loading capacity, stability, sustained release, and ability to penetrate tumour tissues effectively.^16^

Capecitabine (CAP) is an oral prodrug of Fluorouracil that is used to treat various cancers, including breast, colorectal, and gastric cancers.^17^ CAP is a BCS Class-III drug with high solubility but limited permeability and is rapidly absorbed from the gastrointestinal tract. With a short half-life (0–2 h) and a high daily dosage (2.5 g m^−2^), it requires a sustained-release formulation to prolong antitumour activity and reduce toxicity.^18^ However, its poor permeability limits efficient transport to target tissues.^19^

Nanocarriers improve the efficacy of nanomedicines for breast cancer treatment by mitigating adverse effects and delivering precise drug doses to primary and metastatic tumours, including the vasculature, stroma, cancer cells, and immune cells.^20^ To date, only ten nanomedicines have been approved for clinical treatment of breast cancer,^21^ while many others are under trial, including active and non-targeting formulations. However, active-targeting nanomedicines have yet to achieve the anticipated outcomes.^22^ Drugs in nanocarriers are more stable, circulate longer, and accumulate at specific tumour sites due to the EPR effect.^23^ Nanomicelles can passively accumulate in tumour tissues; however, most of the drug is released before receptor-mediated endocytosis.^24^ Recently developed drug delivery techniques target cancer cell surface receptors, presenting a viable alternative to passive targeting.^25^

Cluster determinant 44 (CD44) is a cell surface glycoprotein receptor highly expressed in tumours such as lung, pancreatic, and breast cancers, particularly known in promoting breast cancer metastasis to the liver.^26^ Targeting CD44 for anticancer therapy offers a promising approach. Hyaluronic acid (HA) is a natural and biocompatible polysaccharide that selectively binds to CD44, enabling intracellular drug delivery to cancer cells. HA-based systems are known for their biocompatibility, biodegradability, and non-toxicity, and are ideal platforms for chemotherapy.^27^

The HA-CD44 interaction plays a critical role in cancer proliferation, migration, and growth. HA-coated nanocarriers with robust core–shell structures enable precise drug delivery to tumour sites, minimizing premature drug release into the bloodstream. Thus, many investigations have analysed HA-coated nanocarriers for targeting CD44 over-expressing cancer cells.^28–31^

Moreover, biocompatible natural surfactants offer significant advantages in micelle drug delivery. Saponins are natural biosurfactants found in plants like *Gypsophila*, *Quillaja*, *Saponaria*, and *Glycyrrhiza* species that enhance the solubility and stability of hydrophobic drugs.^32^ As a primary surfactant, saponin promotes micelle formation and improves encapsulation efficiency.^33,34^ Glycerol acts as a co-surfactant to stabilize micelles by reducing interfacial tension, increasing stability, and enhancing drug loading.^35^ The combination of saponin and glycerol makes them ideal for developing micelles for targeted breast cancer therapy. In addition, vitamin-E TPGS stabilizes micelles by enhancing integrity and drug solubility, supporting efficient delivery in cancer therapy.^36,37^

A novel ligand-coated, receptor-driven nanosystem was designed to encapsulate capecitabine for improved breast cancer treatment. The system uses a saponin micelle loaded with CAP and coated with HA to target tumours *via* HA/CD44 receptor binding. The study used a design of experiments (DoE) approach to optimize formulation and processing parameters, evaluating their effects on particle size, entrapment efficiency, and drug release. The nanomicelle structure is expected to accumulate at tumour sites through EPR and be internalized by CD44-overexpressing breast cancer cells. This delivery system aims to induce apoptosis and suppress metastasis.

## Materials

### Chemicals

Capecitabine (CAS Number: 154361-50-9) was obtained from Dr Reddy's Laboratories (Hyderabad, India) as a gift sample. Saponin (ex. *Gypsophila* roots 25% sapogenin; CAS Number: 8047-15-2) and glycerol (CAS Number: 56-81-5) were purchased from Sisco Research Laboratories (SRL) Pvt. Ltd (Maharashtra, India). Vitamin-E TPGS (CAS Number: 9002-96-4) was received as a gift sample from Matrix Life Science Pvt. Ltd (Aurangabad, India). Hyaluronic acid (800 kDa) (CAS Number: 9004-61-9) was purchased from BFC Lab Pvt. Ltd (Bangalore, India). If not specified, distilled filtered water was used and all other organic solvents or reagents were of analytic grade and used as received.

### Cell culture

Human breast cancer cell line MCF-7 was purchased from the National Centre for Cell Science (NCCS), Pune, India. Dulbecco's modified Eagle's medium (DMEM) containing 10% fetal bovine serum (FBS), and 1% penicillin–streptomycin was used to culture MCF-7 breast cancer cells. All cells were incubated at 37 °C in a humidified atmosphere with 5% CO_2_. Trypsin–EDTA, trypan blue, phosphate-buffered saline (PBS), and 3-(4,5-dimethylthiazol-2-yl)-2,5-diphenyltetrazolium bromide (MTT) were used.

## Methods

### Experimental design

Initially, preliminary experiments (one factor at a time approach) were performed to determine the main factors and the appropriate ranges in which the optima lie. Through preliminary screening, the most significant variables were identified, *i.e.*, the effects of concentrations of surfactant (saponin) and co-surfactant (glycerol), and the sonication time as independent variables on the particle size (nm), entrapment efficiency (%) and *in vitro* drug release were tested after 4 h. Based on the preliminary trials, a 3-factor, 3-level Box–Behnken design (BBD) by a response surface methodology (RSM) was applied through the Design-Expert v12.0. Software (Stat-Ease Inc., Minnesota, U.S.A) to study the effect of each independent variable on the dependent variables. For each factor, natural values corresponding to the coded levels of −1, 0, and +1 were selected to cover a range of values of practical interest based on the results of preliminary experiments conducted to assess their effect on the responses (Table S1†). A 3^3^ BBD requires 15 runs with a minimum of three central points to determine the experimental error and precision of the design using RSM. This design is suitable for exploring quadratic response surfaces and constructing second-order polynomial models. It consists of replicated centre points and the set of points lying at the midpoint of each edge of the multidimensional cube that defines the region of interest. The 15 runs were carried out for 3 factors, which were conducted randomly to minimize the effects of uncontrolled factors. Besides, the percentage error was calculated between the observed and expected data. Eventually, the optimized formulation was selected for the next steps.

### Preparation of capecitabine-loaded nanomicelles

**Fig. 1:**
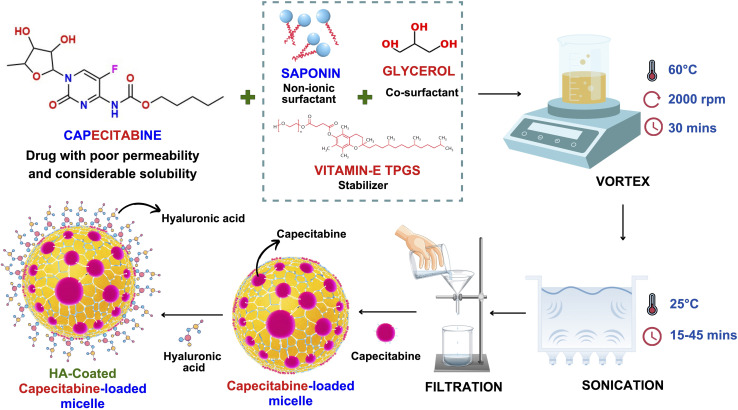
Schematic of the structure of CAP-M and HA-CAP-M nanomicelles and their method of preparation for the CD44 receptor-mediated endocytosis.

The preparation of capecitabine-loaded nanomicelles (CAP-M) was done using the direct dissolution method.^38^ It is the simplest and most feasible method for preparing micelles and is especially appropriate for water-soluble molecules.^39^ This process involves rapid solubilisation of the surfactant and co-surfactant in the aqueous medium followed by steady sonication. Saponin ex. *Gypsophila* roots, glycerol, vitamin-E TPGS, and distilled water were all used for the preparation of nanomicelles. Fifteen formulations of CAP-M were prepared as suggested by the DoE software and were subjected to further characterization. The procedure involved in the preparation of CAP-loaded micelles is illustrated in Fig. 1. Following a preliminary trial, a 3^3^ BBD with 15 formulations was suggested by the Design Expert software (Table 1).

**Table 1:** Design of experiments for the optimization of CAP-M nanomicelle formulation using RSM

Run	Levels of independent variables		
	A: saponin (mmol)	B: glycerol (mmol)	C: sonication time (minutes)
1	+1	0	+1
2	0	+1	−1
3	0	−1	−1
4	0	0	0
5	+1	+1	0
6	+1	0	−1
7	+1	−1	0
8	0	+1	+1
9	−1	0	−1
10	−1	0	+1
11	0	0	0
12	−1	−1	0
13	0	−1	+1
14	0	0	0
15	−1	+1	0

### Preparation and coating of hyaluronic acid on capecitabine-loaded micelles

The optimized CAP-M formulation was coated with hyaluronic acid (HA-CAP-M) by adding 10 mL of 0.1% (w/v) HA solution with stirring at room temperature for 1 h.

### Characterization of capecitabine-loaded micelles

#### Preparation of calibration curve

Capecitabine was diluted in 10 mL of PBS pH 7.4 to make a 1 mg per mL stock solution. Subsequently, the stock solution was diluted to attain concentrations of 10 to 50 μg mL^−1^. UV-visible spectroscopy (V-730 Double Beam, Jasco, Inc., Japan) was used to determine the maximum absorption wavelength by scanning the standard solution against PBS pH 7.4 as blank from 200–400 nm. To develop a calibration curve, absorbance measurements at *λ*_max_ were plotted against the corresponding concentrations using these dilutions. Calibration was validated using the absorption spectrum overlay.^40^

#### ATR-FTIR studies

Fourier Transform Infrared (FTIR) was utilized for determining the drug-excipient compatibility before micelle preparation. The FTIR spectra were acquired using IRTracer-100 (Shimadzu Corporation, Japan) using an attenuated total reflectance (ATR) mode. Individual FTIR spectra were generated for pure CAP, saponin, glycerol, vit-E TPGS, and hyaluronic acid and subsequently compared. Subsequently, 5 mg of each sample was positioned, and scans were conducted with the torque gradually increased up to 70%. The examined infrared spectral wavelength spanned from 4000 cm^−1^ to 400 cm^−1^, with a spectral resolution of 4 cm^−1^.^41^ Similarly, following the preparation of micelles, the FTIR spectra of CAP-M and HA-CAP-M were also analysed and compared with that of the pure drug to ensure that the nature of the drug is retained in the formulations.

#### Tyndall effect

The Tyndall effect is a classic method to qualitatively distinguish between a solution and a micellar suspension.^42^ When a beam of light passes through the micelles, a “bright path” in the colloid can be observed, resulting from the scattering of light by the dispersed particles. This study employed the Tyndall effect to verify the formation of CAP-loaded micelles.

#### Critical micellar concentration

Surface tension measurement at 25 °C determined the CMC values of surfactant (saponin) and co-surfactant (glycerol) producing micelles. This approach used saponin and glycerol solutions of various concentrations. A stalagmometer was used to measure the mass of 50 drops of surfactant and co-surfactant solutions and water. The Hagen–Poiseuille equation was used to calculate the surface tensions of the samples:
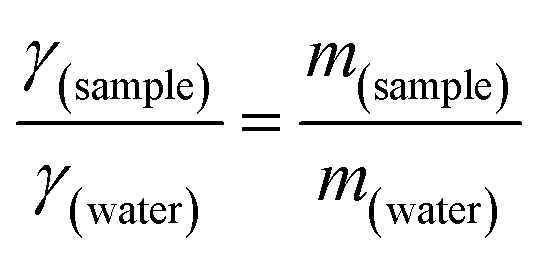
where *γ*_sample_ (N m^−1^) and *γ*_water_ (N m^−1^) denote the surface tension of the surfactant solution and water (at 25 °C), respectively, while *m*_sample_ (g) and *m*_water_ (g) represent the masses of the surfactant solution and that of water. CMC was computed from a plot of concentration *vs.* surface tension. The same approach was used to determine micelle CMC and compare it to the surfactant. A comparison of this CMC value demonstrates the efficacy and stability of micelles, which is essential for the development of a targeted drug delivery system.^43^

#### Particle size, zeta potential, and polydispersity index

The particle size, zeta potential, and polydispersity index (PDI) of the micelle formulations were determined by dynamic light scattering (DLS) at 25 °C *via* the Nano ZS-90 zeta sizer (Malvern Panalytical, UK). A 0.5 mL aliquot of each sample was diluted with distilled water to a final volume of 25 mL before measurement. Zeta potential measurements were evaluated using DLS in conjunction with electrophoretic mobility on the specified equipment.^44^

#### Transmission electron microscopy (TEM) analysis

The particle shape and size were studied using transmission electron microscopy (TEM). Before operating the TEM, the formulated micelle (CAP-M and HA-CAP-M) loaded with the drug were diluted to 0.01% w/w and placed in an ultrasonic bath (GT SONIC-S2 bath ultrasonicator, China) to reduce particle aggregation. The sample was stirred for about 2 minutes and then placed onto a holey carbon-coated 300-mesh copper grid, with the excess suspension immediately removed using filter paper. The grid was kept flat for 20 seconds to allow the colloidal solution to settle and dry completely. The size and shape of the drug-loaded and ligand-coated micelles was analysed using a TEM (JEOL JEM-2100; JEOL Inc., Japan) with an accelerating voltage of 80 kV. The distribution of micelle dimensions was evaluated from TEM images using Radius control & imaging software (EMSIS GmBH, Germany).^45^

#### Entrapment efficiency

The entrapment efficiency (EE%) of CAP-M was determined indirectly by quantifying the non-entrapped drug remaining in the aqueous phase after separating the micelles.^46^ The micellar suspension was centrifuged at 14 000 rpm for 1 h at 4 °C using a refrigerated centrifuge (REMI C-24, Maharashtra, India). The clear supernatant was carefully collected, diluted appropriately with phosphate buffer (pH 7.4), and analysed using a UV spectrophotometer (V-730 Double Beam, Jasco, Inc., Japan) at 304 nm in PBS pH 7.4.

#### *In vitro* drug release study and kinetic modelling

The drug-loaded micelle formulations were evaluated for *in vitro* drug release under sink conditions using dialysis with PBS pH 7.4 as the release medium.^47^ Two mL of sample was placed in a dialysis bag (Mol. Wt. cut-off: 12 000 to 14 000 Da; Dialysis Membrane-60, Himedia, Maharashtra, India), continuously agitated at 50 rpm and maintained at 37 °C in a 50 mL PBS solution (*T* = 37, pH = 7.4). Samples were withdrawn at 0, 1, 2, 4, 6, 8, 12, and 24 h and the amount of drug present was quantified using UV spectroscopy. The procedure was performed in triplicate. To analyse the drug release profile and mechanism, the release data were fitted to various linear kinetic models, including zero-order-, first-order-, Higuchi-, Hixson–Crowell-, and Korsmeyer–Peppas models.

#### *In vitro* permeability studies

The *in vitro* permeation study of capecitabine from micelles was evaluated using a modified Franz diffusion cell with a diffusion area of 4.52 cm^2^. An Isopore™ polycarbonate membrane Filter (0.4 μm) was used as the permeation membrane between the donor and acceptor chambers. The acceptor chamber contained 15 mL of PBS (pH 7.4) to maintain sink conditions, while CAP-M formulation (1 mg per mL capecitabine) was added to the donor chamber. The setup was maintained at 37 ± 0.5 °C with stirring at 100 rpm. At predetermined time intervals (0, 1, 2, 4, 6, 8, 12, and 24 hours), aliquots of samples were withdrawn, replaced with PBS, filtered, and analysed *via* UV-vis spectroscopy (*λ*_max_ = 304 nm). The cumulative drug permeation (*Q*, μg cm^−2^) was plotted over time to calculate flux (*J*, μg cm^−2^ h) from the linear curve slope, and the permeability coefficient (*P*, cm h^−1^) was calculated by dividing the flux by the initial drug load.^48,49^

### Stability studies

#### Storage stability

The stability of the optimized formulations CAP-M and HA-CAP-M was studied at 4 °C and 25 °C for up to 60 days. The stability of formulations and the effect of storage conditions on average particle size, PDI, and EE were assessed after 60 days.^50^

#### Dilution stability

Micelle stability under dilution was studied employing dilution testing. CAP-M and HA-CAP-M were diluted 10, 50, and 100 times with PBS pH 7.4, and the mean particle size, PDI, and zeta potential of the formulations were evaluated.^51^

#### *In vitro* cytotoxicity studies

MTT assay were utilized to analyse cell viability, cytotoxicity, and inhibitory effects of free drug CAP, optimized CAP-M, and HA-CAP-M at a range of concentrations. The MCF-7 cells were seeded at a density of 1000–10,000 cells per well, as 200 μL per well in 96 well plates and cultured in standard 10% DMEM medium at 37 °C overnight to reach confluency. The treatment was carried out by the addition of the above-mentioned samples at various concentrations in the range of (1.5, 3.1, 6.2, 12.5, 25, 50 μM, and μg mL^−1^). The cells were incubated at 37 °C for 24 h. 200 μl of MTT working solution was added and the plates were incubated in absolute darkness at 37 °C for 4 h. To dissolve the purple formazan crystals, 1 mL of DMSO/well was added and the absorbance was measured at 590 nm using a spectrophotometer.^52^

#### Acridine orange/propidium iodide staining assay

Acridine orange (AO)/propidium iodide (PI) staining is a crucial technique for the detection of apoptosis. When the AO dye permeates the entire cell membrane, it causes the nuclear DNA to appear fluorescent green, whereas the PI stain exhibits red-orange fluorescence in the DNA of damaged cells. In this study, the cells were seeded at a density of 1 × 10^6^ cells per well in a six-well plate. After 24 h of incubation, the old medium was replaced with fresh media containing the drug. After 24 h of incubation, the cells were washed with PBS and treated with a mixture of 10 μg per mL AO and 10 μg per mL PI (dissolved in PBS). Thereafter, the cells were examined under a fluorescence microscope.^53^

## Results

### Pre-formulation studies

#### Preparation of calibration curve

**Fig. 2:**
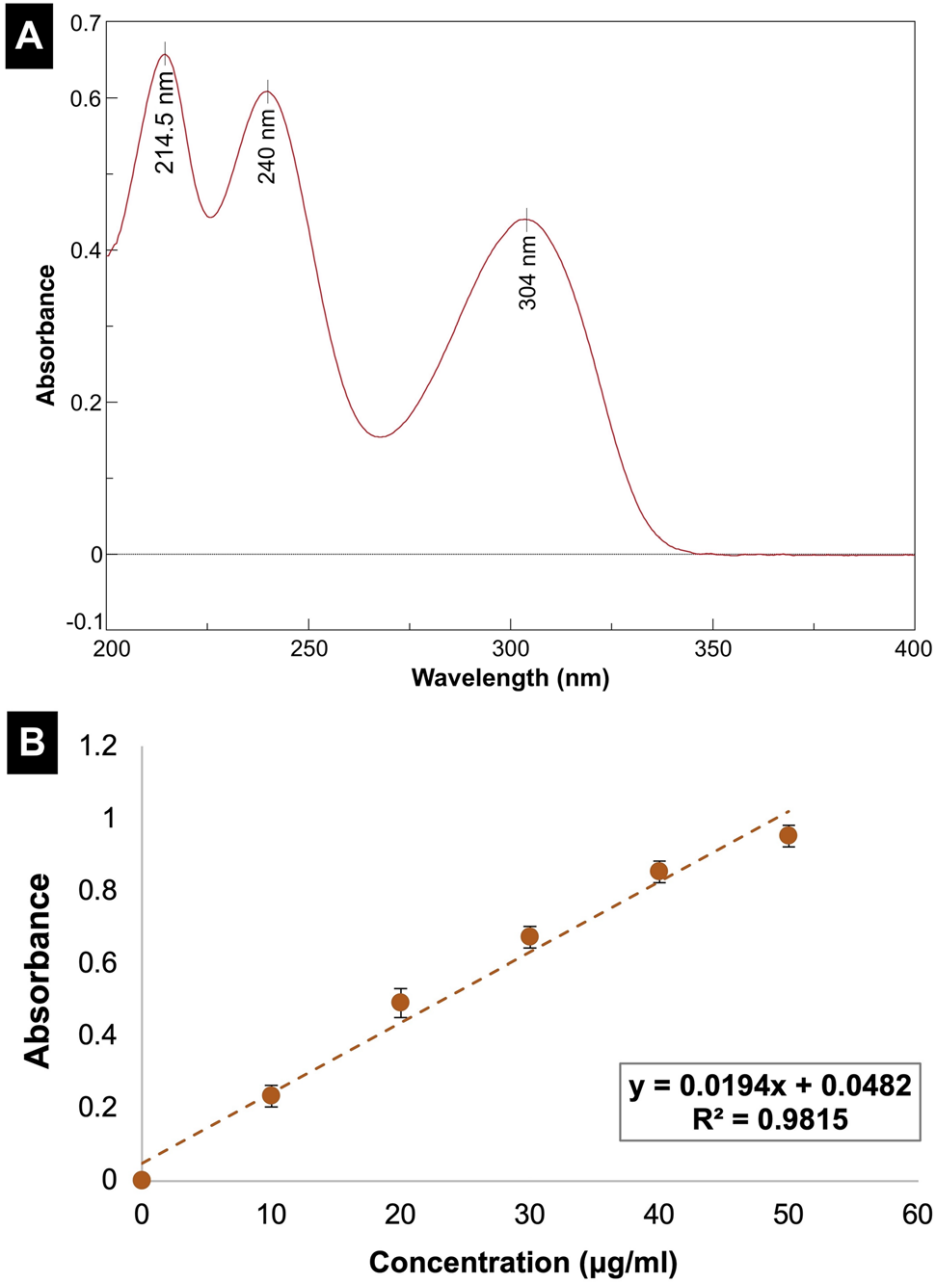
(A) UV/vis spectrum and (B) calibration curve of capecitabine in PBS pH 7.4.

The standard drug solution of CAP exhibited significant absorbance peaks at 214.5 nm, 240 nm, and 304 nm in UV-vis spectroscopy. Based on the peak characteristics and supporting existing literature,^54,55^ 304 nm was identified as the *λ*_max_ for capecitabine. A calibration curve was constructed to evaluate the linear relationship between the drug concentration and its optical density. A strong linear correlation between concentration and absorbance was achieved within the range of 10 to 50 μg mL^−1^. The UV spectrum and calibration curve of capecitabine are shown in Fig. 2.

#### Drug-excipient compatibility studies

**Fig. 3:**
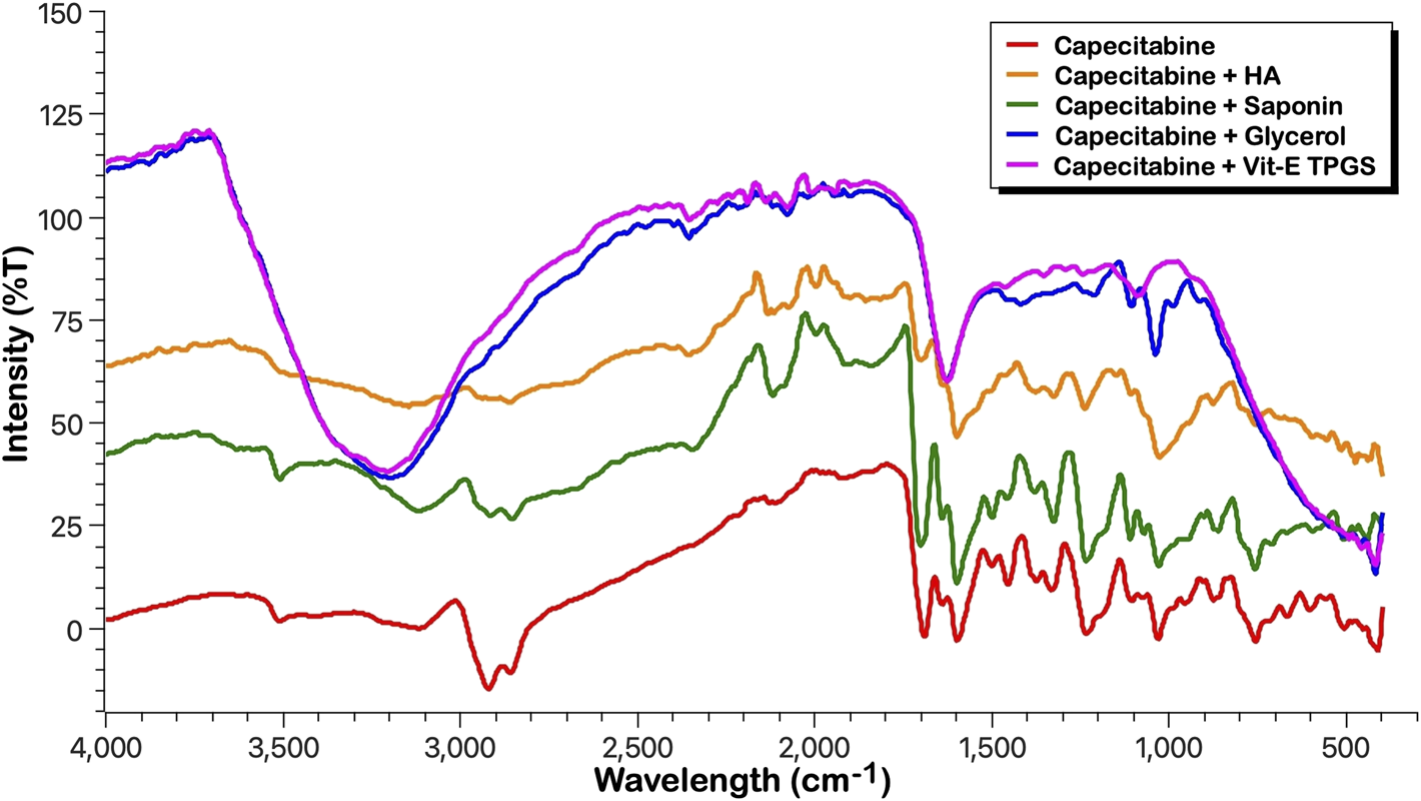
Stacked FTIR spectra of capecitabine and excipients (HA, saponin, glycerol and vit-E TPGS).

Fig. 3 compares the FTIR spectra of capecitabine with different excipients in the formulation, including HA, saponin, glycerol, and vit-E TPGS. The spectrum of pure CAP shows distinct characteristic peaks, such as the N–H stretching (amide) at 3510.45 cm^−1^, C=O stretching (ester and amide) at 1641.42 cm^−1^, aromatic C=C stretching at 1454.33 cm^−1^, and C–F vibrations at 1078.21 cm^−1^. These significant peaks are preserved in the spectra of the formulations with different excipients, indicating that no chemical interaction or incompatibility occurred between CAP and the excipients. The slight variations in intensity are attributed to physical effects such as dilution or blending rather than chemical modification.

The absence of new peaks, disappearance of critical functional group peaks, or significant shifts confirms the compatibility between CAP and the tested excipients. Each formulation retains the structural integrity of CAP, as evidenced by the consistent presence of its key functional groups. This suggests that the excipients do not chemically react with CAP, supporting the stability of the drug in these formulations.

### Characterization of capecitabine-loaded micelle formulation

#### FTIR analysis

**Fig. 4:**
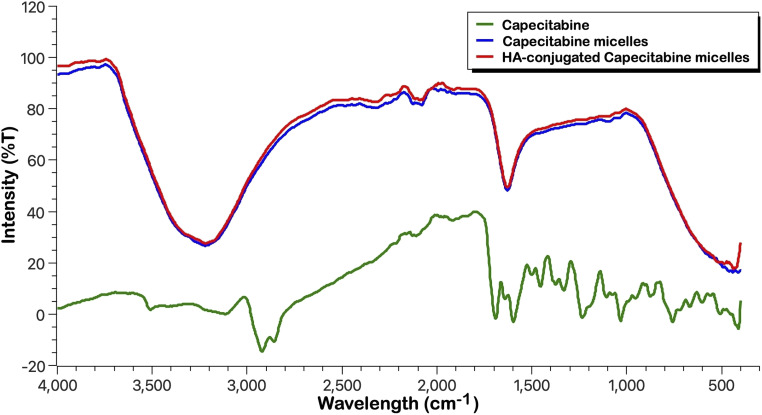
FTIR spectra of capecitabine, CAP-M and HA-CAP-M.

Following the preparation of formulation, the formulations loaded with capecitabine with and without HA coating (CAP-M and HA-CAP-M) were analysed by ATR-FTIR, and the spectra of the formulations were studied in comparison with that of the free drug (Fig. 4). CAP-M and HA-CAP-M spectra closely align with the CAP spectrum, exhibiting key functional groups identical to its structure, showing no new peaks, significant shifts, or loss of functional group peaks. This demonstrates that the micelle formulations do not chemically alter or degrade capecitabine.

The preservation of capecitabine's structural peaks in the micelle formulations confirms the stability and integrity of the drug. The formulations encapsulate the drug without inducing any chemical interaction, as evidenced by the consistent FTIR profiles. This suggests that the micelles are effective in maintaining the drug's chemical nature and stability during formulation. Fig. S1–S7 and Table S2† show the results and interpretation of the FTIR analysis.

#### Tyndall effect

**Fig. 5:**
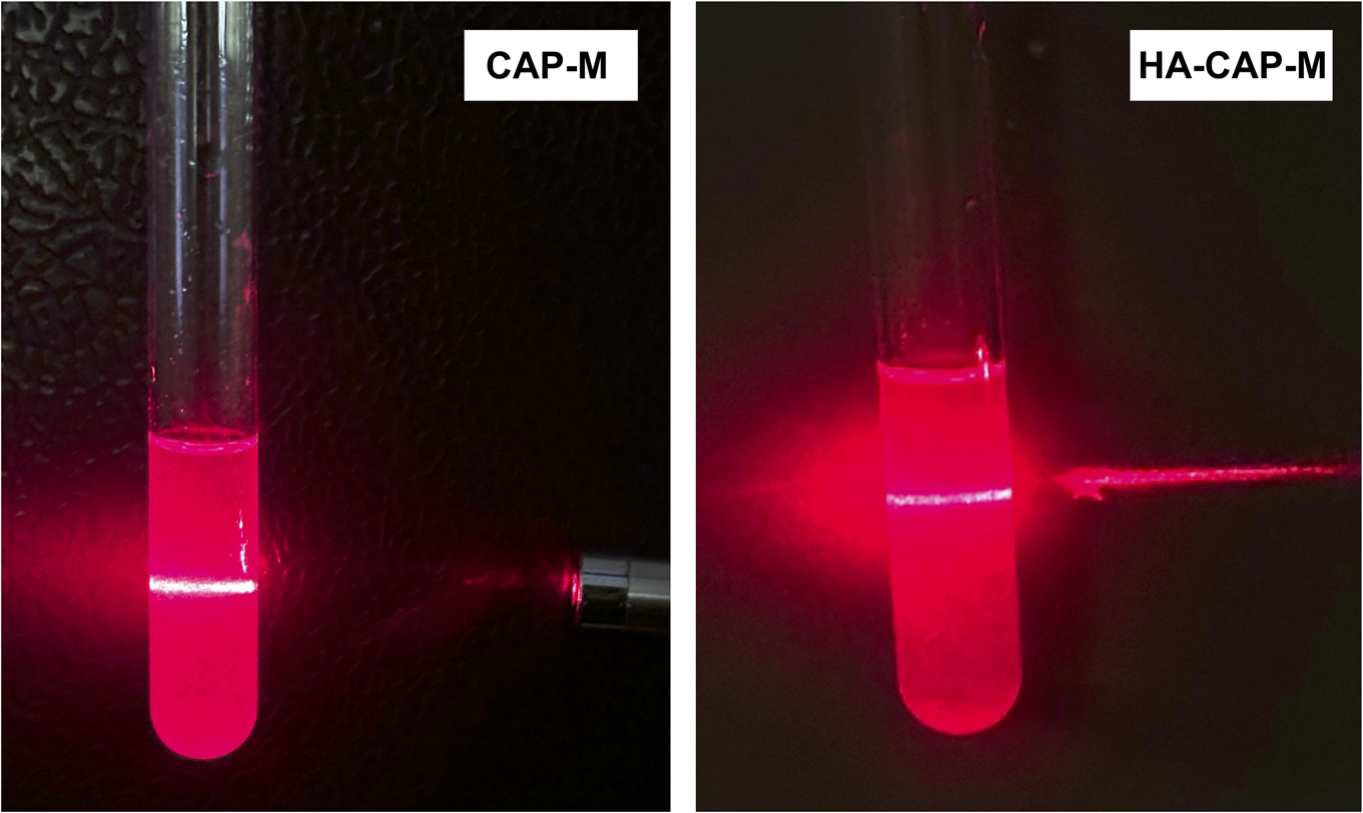
Tyndall effect exhibited by CAP-M and HA-CAP-M.

CAP-loaded nanomicelles prepared by the aforementioned method were clear, transparent, and mildly frothy at room temperature. A “bright pathway” was observed when a micellar suspension was laser-irradiated, which is attributed to the Tyndall effect (Fig. 5). This test confirmed the formation of micelles in the formulation.

#### Critical micellar concentration

**Fig. 6:**
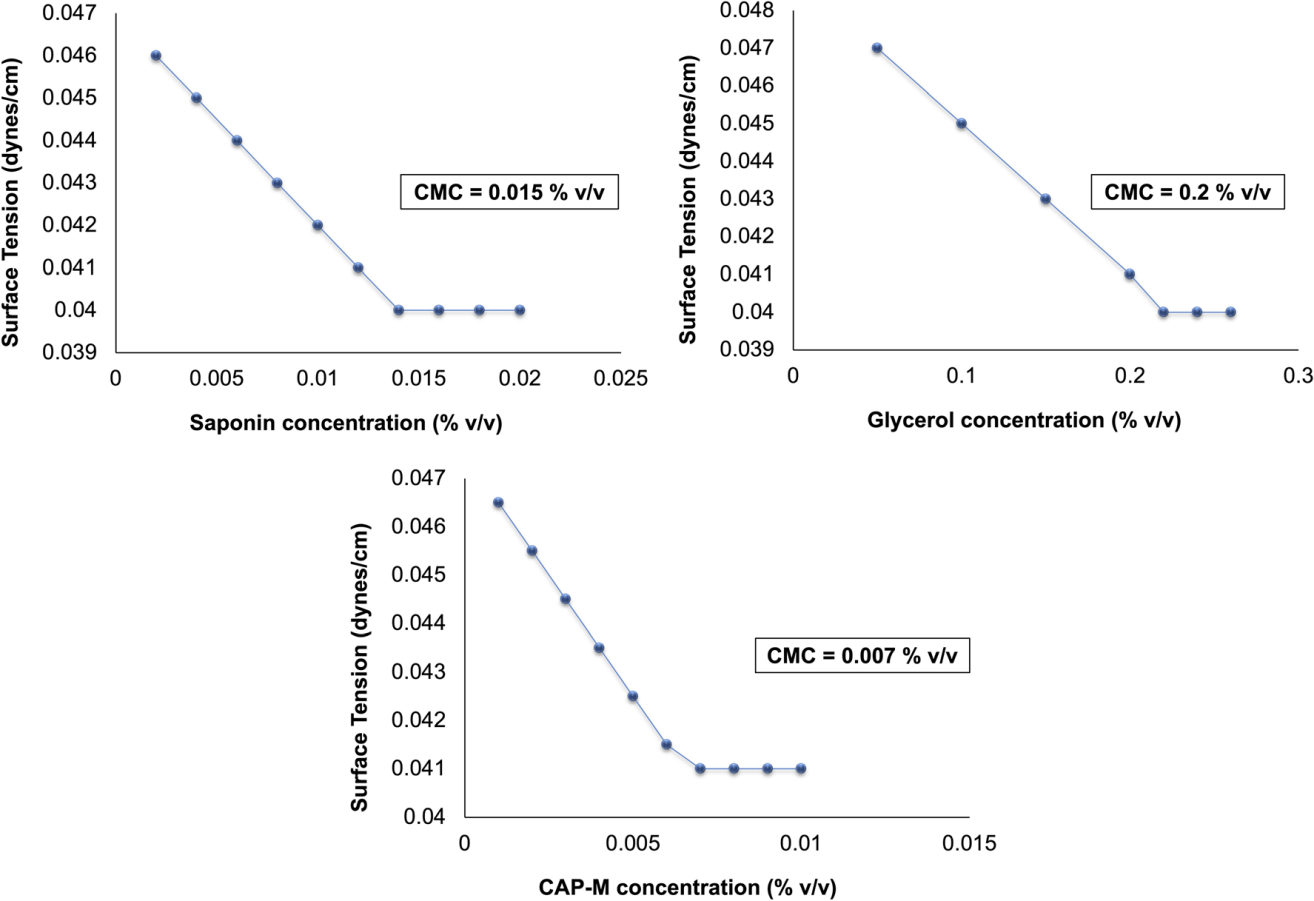
CMC of saponin, glycerol and CAP-M.

The CMC of the surfactant saponin, the co-surfactant glycerol, and the capecitabine-loaded micelle (CAP-M) were determined by analysing the changes in surface tension as a function of concentration (Fig. 6). The CMC of saponin was 0.015% (v/v), where the surface tension reached a plateau, signifying the initiation of micelle formation as the surfactant molecules transitioned from reducing surface tension to forming micelles. In comparison, glycerol exhibited a higher CMC of 0.2% (v/v), reflecting its relatively weaker surface activity as a co-surfactant.

Upon incorporating CAP into the micellar system, an approximately 2.5 decrease in the CMC was observed compared with the CMC of saponin. Comparable findings in other studies^56,57^ highlight the combined effect of the components on drug encapsulation and micelle formation. The drug–surfactant interactions likely enhance the thermodynamic stability of the micelles, facilitating their formation at much lower surfactant concentrations. The marked reduction in the CMC of the CAP-M demonstrates its superior surface activity and underscores the enhanced stability and compactness of the micelles. These characteristics are particularly advantageous in pharmaceutical formulations as they ensure efficient micellization and drug loading at reduced surfactant concentrations.^58^

#### Optimization of capecitabine-loaded micelles

**Fig. 7:**
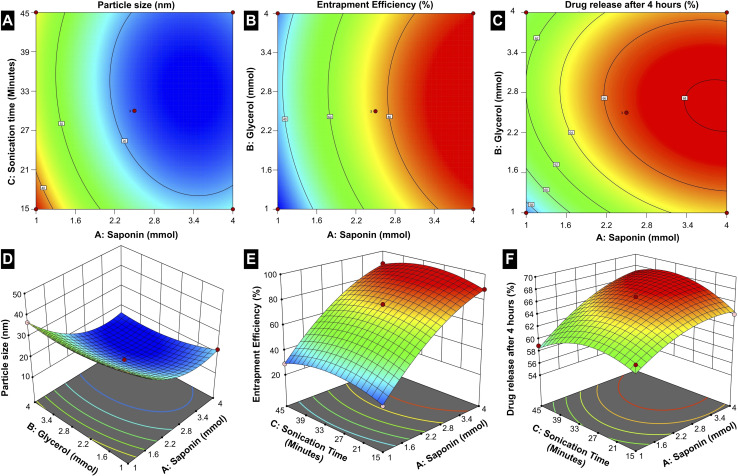
Box–Behnken design for (A and D) particle size, (B and E) entrapment efficiency and (C and F) *in vitro* drug release after 4 h as a function of the parameters (surfactant and co-surfactant concentration, and sonication time).

In Box–Behnken design, optimal responses are suggested by calculating the effect of each independent factor and their interactions.^59^ The selected independent variables were surfactant concentration, co-surfactant concentration, and sonication time. The subsequent results of particle size, EE, and *in vitro* drug release after 4 h were used for optimization studies. Table 2 illustrates the results of the optimization tests. The size and EE of CAP-M ranged from 14.92 to 44.52 nm and 23.75 to 89.3%, respectively (Table 2). The % CDR of CAP-M after 4 h ranged from 55.45% to 67.24% (Table 2). The ANOVA for size, EE, and *in vitro* drug release is presented in Table S3.† All responses were fitted to quadratic models and were polynomial (Table S4†). Statistical analysis reveals that the particle size, entrapment efficiency (EE%), and drug release after 4 h are significantly influenced by the independent variables: saponin concentration (A), glycerol concentration (B), and sonication time (C) (Fig. 7).

**Table 2:** Optimization results of CAP-M using the design of experiments by RSM^a^

Run	Particle size (nm)	Entrapment efficiency (%)	Drug release after 4 h (%)
1	19.47 ± 1.43	89.3 ± 2.65	65.82 ± 4.53
2	25.89 ± 1.03	62.11 ± 3.22	60.36 ± 3.22
3	29.42 ± 0.53	56.2 ± 1.89	61.08 ± 2.64
4	17.81 ± 1.54	75.01 ± 4.53	66.52 ± 2.07
5	14.92 ± 2.03	87.97 ± 2.43	67.24 ± 3.04
6	23.82 ± 0.04	88.43 ± 2.11	64.07 ± 2.53
7	21.94 ± 0.42	85.24 ± 1.53	64.75 ± 1.53
8	22.69 ± 1.52	63.29 ± 1.11	62.48 ± 4.24
9	44.52 ± 1.68	28.19 ± 2.59	61.74 ± 2.67
10	35.41 ± 2.53	29.02 ± 2.31	58.94 ± 2.54
11	17.27 ± 2.11	76.92 ± 1.96	66.69 ± 3.25
12	42.08 ± 1.80	23.75 ± 1.04	55.45 ± 4.62
13	24.28 ± 0.98	55.45 ± 1.54	61.2 ± 2.34
14	17.35 ± 1.04	76.73 ± 2.45	66.87 ± 3.24
15	36.82 ± 1.66	32.93 ± 1.42	60.34 ± 2.89

a Values are presented as means ± SD (*n* = 3).

The particle size is notably dependent on all three factors, with the contour and 3D surface plots demonstrating a reduction in size at optimized combinations of A, B, and C. This indicates that precise adjustments in the formulation components and processing parameters are essential to achieving smaller, more uniform micelles, which are critical for effective drug delivery. Entrapment efficiency is primarily governed by A and B, where increasing the saponin concentration and optimizing the glycerol concentration yield higher drug encapsulation. This suggests that the amphiphilic nature of saponin plays a crucial role in creating a stable micellar structure capable of efficiently entrapping the drug, while the co-surfactant glycerol facilitates the stabilization of the micelle. Interestingly, sonication time (C) has less influence on the EE, suggesting that encapsulation is mainly determined by the surfactant-*co*-surfactant interactions rather than the mechanical energy imparted during sonication.

The drug release behaviour is distinctly influenced by A and C; the higher concentration of saponin changes the drug release rates, which is because of a favourable influence on solubilizing the drug in the micelles, while optimized sonication time allows the proper formation of the micelles and stability. Thus, the interplay of these conditions and formulation components outlines the complexities encountered when achieving drug release control. While saponin concentration (A) exerts the most pervasive influence on all three responses among the three factors, glycerol concentration (B) mostly affects particle size and EE. On the other hand, sonication time (C) is crucial for optimizing particle size and drug release, underscoring its role in the physical structuring and functional performance of the micelles.

The statistical evaluation further supports these findings, with *R*-squared and adjusted *R*-squared values closely aligning, indicating a reliable model capable of accurately predicting the responses within the design space (Table S5†). The adequate precision values that measure the signal-to-noise ratio exceed the favourable threshold of 4 for all responses, confirming the robustness of the model. Further, 2.5 mmol of surfactant, 2.5 mmol of co-surfactant, and a sonication time of 30 minutes yields optimum formulations (CAP-M) in terms of particle size, EE, and CDR after 4 h. The values of optimized responses generated from the RSM method and their experimental data under optimal conditions are given in Table S6.†

As seen in the plots, increasing saponin concentration (A) from 1 to 4 mmol initially reduces particle size and then slightly increases it beyond the optimized level. Entrapment efficiency (EE%) follows a similar trend, peaking at intermediate concentrations, while drug release consistently increases with higher saponin levels. Glycerol concentration (B) stabilizes particle size and EE at optimized levels but plateaus at higher values. Meanwhile, sonication time (C) will significantly reduce the particle size with longer durations and facilitate drug release by enhancing micelle stability and uniformity. Moreover, trends relating to sonication and micelle performance emphasize the position of formulation variables. These results describe the need for an optimized formulation, in which a careful balance between the factors is essential to provide the desired responses. Such an in-depth understanding of factor-response relationships forms a sound basis for the rational design of micellar drug delivery systems.

#### Particle size, zeta potential, and polydispersity index

**Fig. 8:**
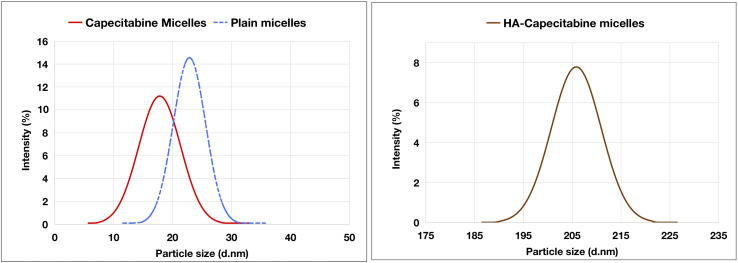
Graphical presentation of the size distribution of plain micelles, CAP-M and HA-CAP-M (mean ± SD (*n* = 3)).

The particle size distribution of plain micelles, CAP-M, and HA-CAP-M is displayed in Fig. 8. The average size of plain micelles, CAP-M, and HA-CAP-M were 23.12 ± 1.14 nm, 17.8 ± 0.75 nm and 205.83 ± 2.04 nm, respectively. In addition, PDI for plain micelles, CAP-M, and HA-CAP-M were 0.282 ± 0.021, 0.277 ± 0.018, and 0.293 ± 0.032, respectively.

**Fig. 9:**
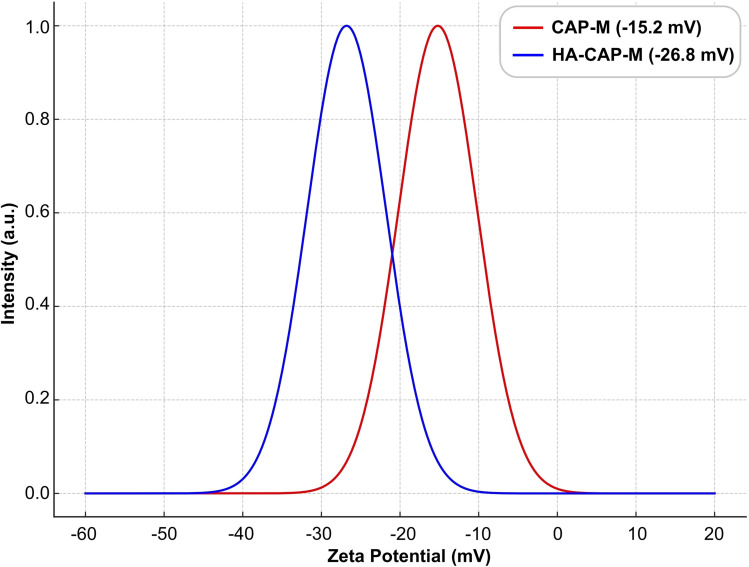
Graphical presentation of the zeta potential distribution of CAP-M and HA-CAP-M (mean ± SD (*n* = 3)).

CAP-M micelles exhibit an average zeta potential of about −15.2 mV (Fig. 9), indicating moderate stability, while the HA-CAP-M micelles have a more negative average zeta potential of −26.8 mV, suggesting enhanced stability due to stronger electrostatic repulsion. The shift in zeta potential reflects surface modifications induced by HA coating. The particle size range aligns with reported ranges, supported by literature attributing them to the core–shell structure and HA coating of the micelles.^60,61^

#### Transmission electron microscopy (TEM) analysis

**Fig. 10:**
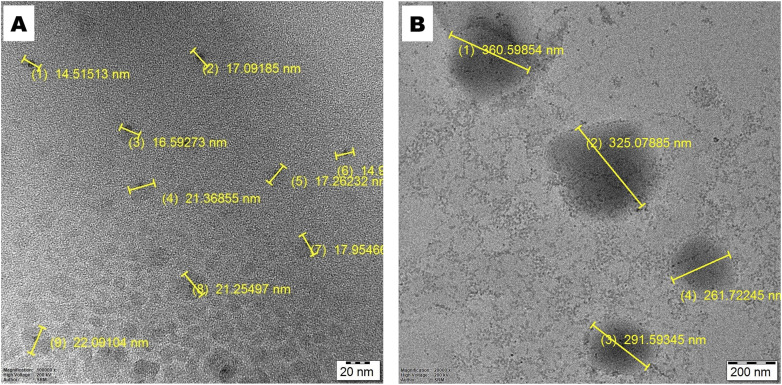
TEM images of (A) CAP-M and (B) HA-CAP-M.

The size and shape of CAP-M and HA-CAP-M were analysed using TEM (Fig. 10). TEM images revealed uniform particle dispersity and spherical structures of the micelles. The HA-coated micelles (HA-CAP-M) were notably larger than the uncoated micelles (CAP-M), consistent with the contribution of the ligand-coating leading to increased size. The micelles exhibited size ranges of 10–50 nm for CAP-M and 180–360 nm for HA-CAP-M, confirming their nanoscale dimensions.

Additionally, TEM results highlighted the spherical shape and uniformity of the micelles. The results were consistent with the findings of TEM analysis in previously reported micelle formulations.^62^ The findings emphasize that HA coating significantly increases the size of the micelles while maintaining their spherical structure and uniform distribution.

#### *In vitro* drug release studies and kinetic modelling

**Fig. 11:**
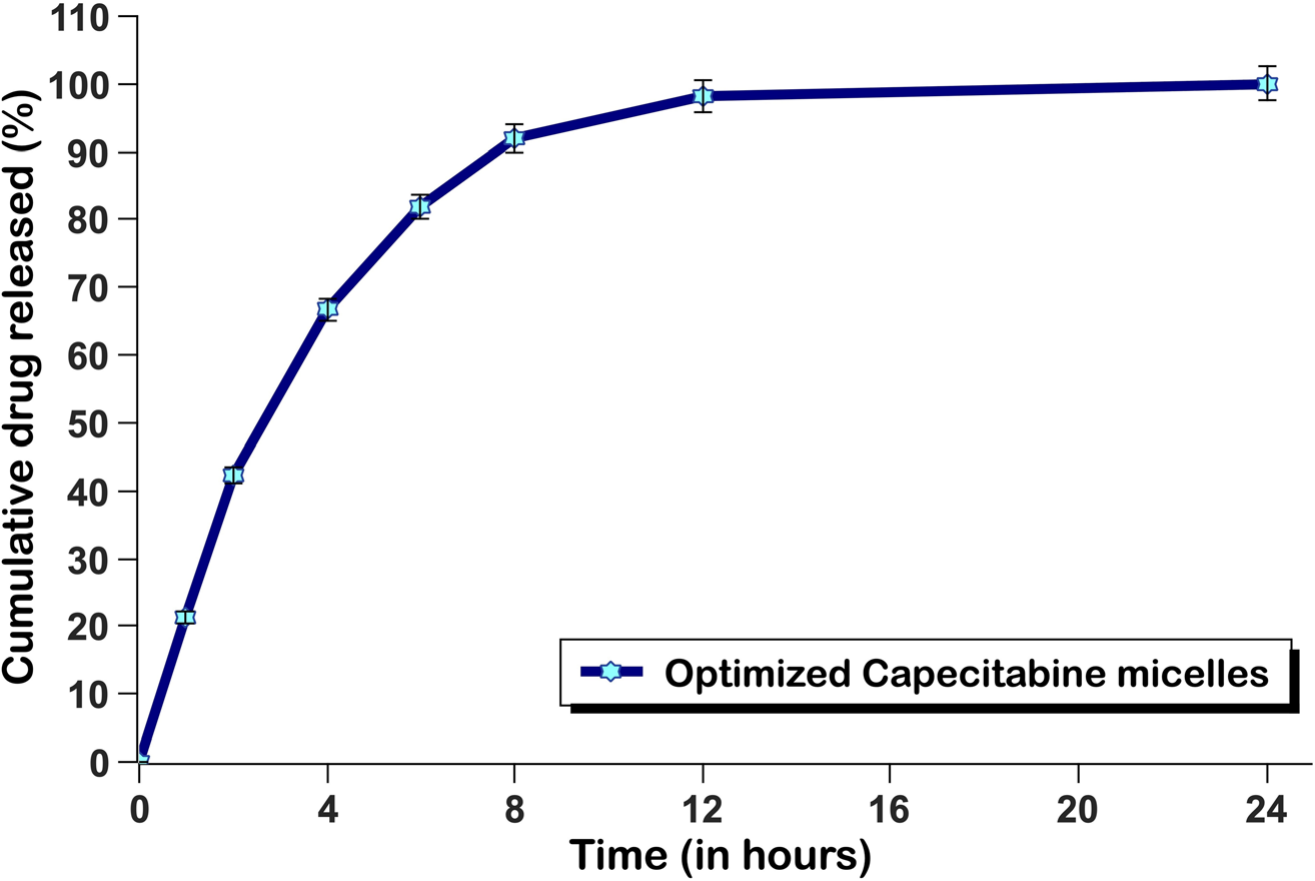
*In vitro* drug release profile of the optimized CAP-M (*n* = 3).

The *in vitro* drug release profile of the optimized CAP-M over 24 h follows a biphasic pattern (Fig. 11), characterized by an initial rapid drug release phase that can be attributed to the diffusion of the drug from the micelle surface, followed by a sustained release phase. Approximately 50–60% of the drug is released within the first 8 h, indicating effective immediate drug availability. This was followed by a slower release phase, causing extended drug release, probably controlled by matrix degradation and drug diffusion from the micelles. This suggests that these micelles have the potential to perform sustained drug release, enabling controlled delivery and prolonged release, thus reducing the frequency of dosing and potentially increasing the efficacy of the treatment.

Release kinetics were analysed by fitting the formulations to various mathematical models. Regression coefficient values are summarized in Table 3. The highest value of the coefficient of determination (*R*^2^ = 0.9179) was given by the Hixson–Crowell model, indicating that this model was the most suitable to describe the drug release because it is predominantly controlled by surface area and/or particle size change of the micelles. The Korsmeyer–Peppas model yields an *R*^2^ value of 0.8655, with release exponent (*n* = 0.49) indicating that drug release is primarily governed by anomalous diffusion, which incorporates both diffusion and erosion. Thus, it seems that the actual release mechanism is complex and involves a combination of diffusion and surface erosion processes as characterized by the Hixson–Crowell and anomalous diffusion models.

**Table 3:** Kinetic models and calculated regression coefficients of drug release from CAP-M

Model	Equation	*R* ^2^
Zero-order	*C*_*t*_ = *C*_0_ − *K*_0_·*t*	0.5907
First order	ln *C*_*t*_/*C*_0_ = −*k*_1_·*t*	0.8857
Higuchi	*M*_*t*_/*M*_∞_ = *Kt*^*n*^	0.8534
Hixson–Crowell	*W*_0_^1/3^ − *W*_*t*_^1/3^ = *k*_*t*_·*t*	0.9179
Korsmeyer–Peppas	*C* = *K*_H_·√*t*	0.8655 (*n* = 0.49)

These findings are consistent with previous studies. For instance, the Hixson–Crowell model describes drug release from systems where there is a change in surface area and diameter of particles or tablets, which aligns with the observed release behaviour in micellar systems.^63^ Similarly, the Korsmeyer–Peppas model has been utilized to study drug release from liposomes, indicating that drug release is primarily governed by anomalous diffusion, which incorporates diffusion and erosion mechanisms.^64^ Therefore, the observed biphasic release pattern and the applicability of these models to describe the release kinetics are well-supported by existing literature.

#### *In vitro* permeability studies

**Fig. 12:**
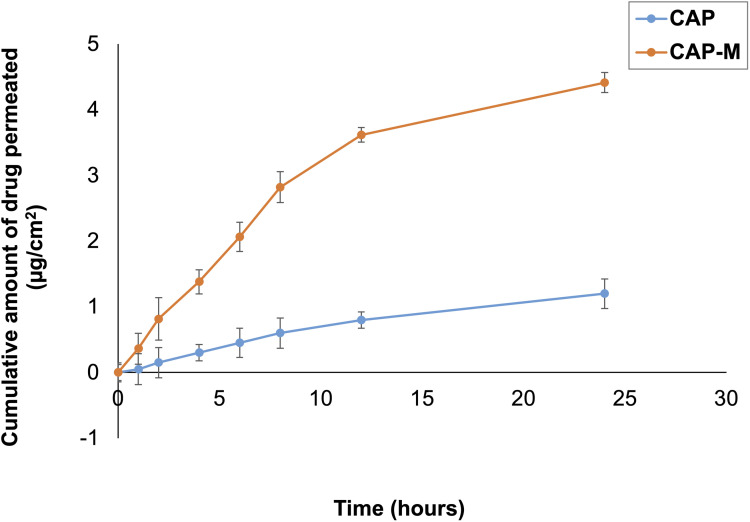
*In vitro* permeation profile of capecitabine and CAP-M formulation. Values are presented as means ± SD (*n* = 3).

The *in vitro* permeation study was conducted utilizing the optimized CAP-M formulation (Fig. 12). The permeation graph was plotted between the cumulative drug permeated (μg cm^−2^) and time (h) and the permeation parameters were calculated such as the rate of drug permeation at steady state (*J*_ss_) or flux, and the permeability coefficient (*K*_p_). Table 4 displays the *J*_ss_ and *K*_p_ values for the CAP-M formulation and the pure drug CAP.

**Table 4:** Permeability parameters for CAP and CAP-M^a^

Formulation	*J*_ss_ or flux (μg cm^−2^ h^−1^)	*K*_p_ (cm h^−1^)
Pure drug capecitabine	0.075 ± 0.015	0.0100 ± 0.0020
Optimized Cap-M	0.391 ± 0.017	0.0521 ± 0.0023

a Values are presented as means ± SD (*n* = 3).

The *in vitro* permeation profile of the optimized CAP-M formulation, comprising saponin and glycerol micelles, demonstrated a significant enhancement in CAP permeation compared to the pure drug CAP. This finding aligns with previous research indicating that saponin-based micelles can effectively increase the solubility and permeability of poorly soluble drugs. For instance, a study utilizing saponin micelles for budesonide delivery reported improved mucosal permeation and a faster onset of action, highlighting the potential of saponin micelles in enhancing drug absorption.^65^ This improvement can be attributed to the ability of micelles to solubilize hydrophobic drugs and modify the barrier properties of biological membranes, thereby enhancing drug transport.

### Stability studies

#### Storage stability

**Fig. 13:**
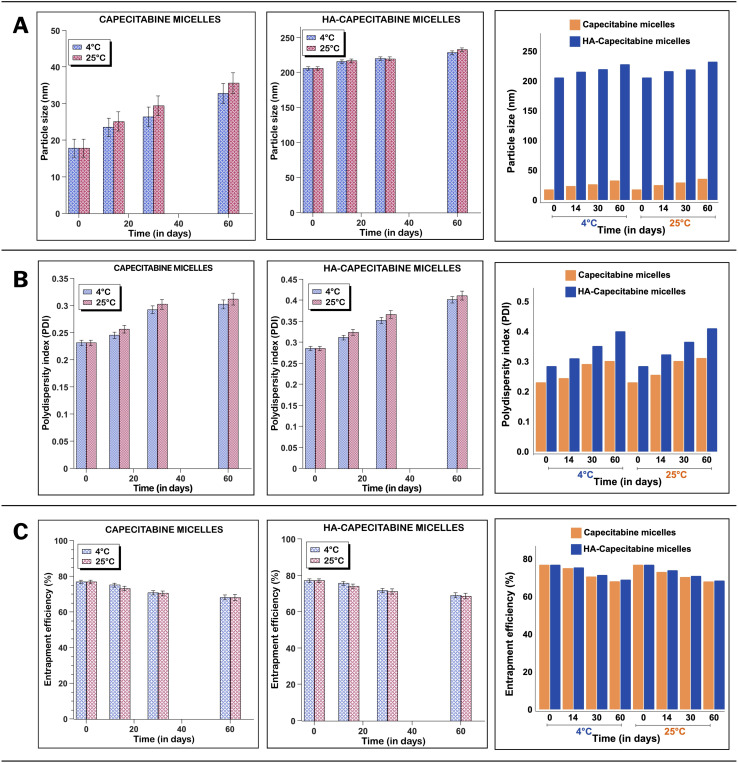
Storage stability of CAP-M and HA-CAP-M stored at 4 °C and 25 °C for 2 months. (A) Particle size (nm). (B) PDI. (C) EE (%). Values are presented as means ± SD (*n* = 3).

The size and PDI showed an increasing trend with time and temperature (Fig. 13). Therefore, the stability of the optimized formulation stored at 4 °C was higher than those held at 25 °C. The micelles at different temperatures showed no substantial decline in EE% over the first two weeks. Nonetheless, significant differences were observed at the 30-day and 60-day marks for both groups, although the HA-CAP-M formulation showed a relatively smaller reduction.

#### Dilution stability

After injection into the bloodstream, drug-encapsulated micelles disperse several times and dissociate into individual monomers. Dilution in water by 5-, 10-, or 100-fold did not appreciably alter the size of the micelles (Table 5). Such findings indicated that CAP-M and HA-CAP-M would be more resilient to dilution following administration.

**Table 5:** Dilution stability of CAP-M and HA-CAP-M^a^

Formulation	Particle size (nm)		
	Dilution volume 5 mL	Dilution volume 10 mL	Dilution volume 100 mL
CAP-M	18.01 ± 0.32	22.15 ± 0.96	25.32 ± 1.42
HA-CAP-M	208.73 ± 0.63	212.67 ± 0.55	214.11 ± 0.05

a Values are presented as means ± SD (*n* = 3).

#### *In vitro* cytotoxicity studies

**Fig. 14:**
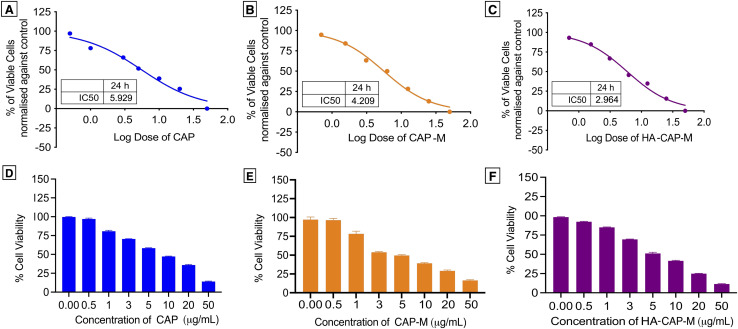
Cytotoxicity of CAP, CAP-M and HA-CAP-M against the MCF-7 cell line by MTT assays. IC50 values and % cell viability of (A and D) capecitabine, (B and E) CAP-M and (C and F) HA-CAP-M.

The *in vitro* cytotoxicity of CAP, CAP-M, and HA-CAP-M was evaluated using the MTT assay against MCF-7 cells over 24 h. A dose-dependent reduction in cell viability was observed across all the test groups, emphasizing their capability to suppress cancer cell proliferation effectively (Fig. 14).

Free CAP exhibited an IC_50_ value of 5.929 μg mL^−1^, reflecting its potent anti-cancer activity. However, this also highlights its limitations, including poor permeability and limited intracellular uptake. The CAP-M micellar formulation demonstrated improved cytotoxicity with an IC_50_ value of 4.209 μg mL^−1^, indicating its ability to enhance drug solubility and stability. This improvement is attributed to the micelles' capacity to facilitate intracellular delivery *via* passive targeting mechanisms, such as the EPR effect. The most significant enhancement in cytotoxicity was observed with the HA-CAP-M formulation, which displayed an IC_50_ of 2.964 μg mL^−1^, the lowest among the three formulations. The superior efficacy of HA-CAP-M can be attributed to the active targeting properties provided by the HA coating. HA selectively binds to CD44 receptors, which are overexpressed on breast cancer cells, enabling receptor-mediated endocytosis and improving intracellular drug accumulation. This targeted delivery combines the benefits of micelle-mediated solubilization and stabilization with active receptor targeting.

HA-conjugated nanocarriers can improve drug accumulation in tumour cells while reducing off-target effects, as demonstrated in breast cancer models.^66,67^ Additionally, micelle-based formulations enhance the solubility and stability of poorly soluble drugs and exploit the EPR effect for passive tumour targeting.^68^ The observed reduction in IC_50_ values for HA-functionalized micelles aligns with previous studies, confirming the synergistic benefits of combining passive micellar delivery with active receptor targeting for effective and selective cancer therapy. Overall, CAP-HA-M exhibited the most improved cytotoxicity compared to CAP and CAP-M, combining enhanced solubility, passive EPR targeting, and active CD44 receptor-mediated targeting. These findings underscore the potential of HA-functionalized micelles as a promising strategy for targeted breast cancer therapy, offering a more effective and selective delivery system for capecitabine.

#### Apoptosis detection by AO/PI dual staining

The AO/PI staining assay results demonstrate a dose-dependent cytotoxic effect of HA-CAP-M against MCF-7 breast cancer cells. MCF-7 cells were treated with different concentrations of HA-CAP-M (6.25, 12.5, 25, and 50 μg mL^−1^). The cells were treated with concentrations higher than the IC_50_ value of the formulation. The fluorescence micrographs were morphologically analysed such that live, dead, and apoptotic cells could be distinguished. AO penetrates both live and dead cells and gives green fluorescence to the nuclei of these cells whereas PI only penetrates dead nucleated cells with compromised nuclear material and stains them red. Thus, dead or necrotic cells give red fluorescence, viable cells green, late apoptotic cells orange, and early apoptotic cells a softer yellow-orange fluorescence.

**Fig. 15:**
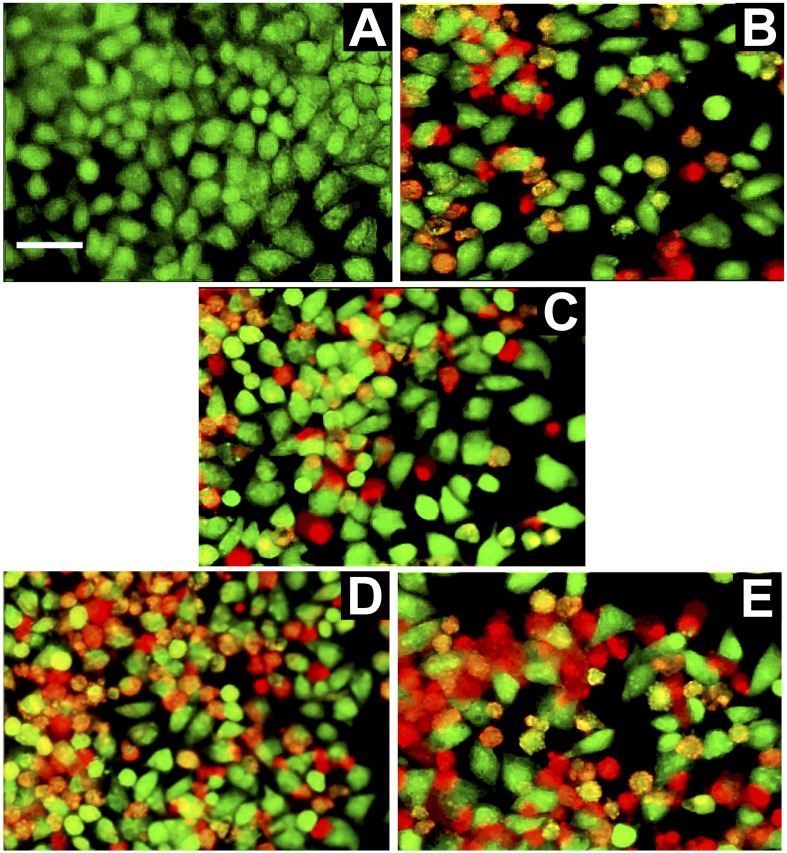
Fluorescence micrographs of MCF-7 cells double-stained with AO/PI. Cells were treated with (A) control and (B–E) HA-CAP-M at 6.25, 12.5, 25, and 50 μg mL^−1^, respectively.

Fig. 15 shows the AO/PI staining fluorescence micrographs of MCF-7 cells. The control group (Fig. 15A) showed predominantly green fluorescence, indicating healthy, viable cells with intact membranes. A slight increase in orange/red fluorescence was observed at 6.25 μg mL^−1^ (Fig. 15B), suggesting limited apoptosis or necrosis. As the concentration increased to 12.5 and 25 μg mL^−1^ (Fig. 15C and D), a higher proportion of apoptotic and necrotic cells was evident, with a noticeable decline in green fluorescence. Red fluorescence dominated at the highest concentration (50 μg mL^−1^, Fig. 15E), indicating extensive cell death through late apoptosis and necrosis.

These findings highlight the potent, dose-dependent cytotoxicity of HA-CAP-M, likely due to its ability to target CD44 receptors on MCF-7 cells, thereby enhancing drug delivery and inducing cell death effectively. The literature strongly supports the findings of dose-dependent cytotoxicity observed with HA-CAP-M against MCF-7 cells using AO/PI staining. Hyaluronic acid-functionalized drug delivery systems have been well-documented to effectively target CD44 receptors, which are overexpressed on breast cancer cells, enhancing intracellular drug delivery *via* receptor-mediated endocytosis.^69^

The differential staining observed in AO/PI assays is a widely recognized method for distinguishing live-, apoptotic-, and necrotic cells, with studies reporting similar fluorescence patterns in dose-dependent cytotoxicity assessments for HA-conjugated formulations.^70^ Furthermore, the increased induction of apoptosis and necrosis at higher concentrations aligns with previous findings demonstrating that HA-functionalized micelles enhance therapeutic efficacy by promoting targeted delivery and inducing significant cancer cell death.^71,72^ These results reinforce the potential of HA-CAP-M as an effective, receptor-targeted therapeutic for breast cancer treatment.

## Discussion

This study focuses on the development and evaluation of hyaluronic acid-coated capecitabine-loaded nanomicelles for CD44 receptor-mediated breast cancer therapy. capecitabine is extensively used for treating numerous cancers including breast cancer. The intrinsic limitations of CAP, such as low permeability, short half-life, and high dosage requirements, necessitate developing a novel delivery system. To overcome these limitations, the current study aims to increase the therapeutic effectiveness and bioavailability of CAP by utilizing nanotechnology and active targeting mechanisms. Nanomicelles have been extensively reported as effective drug carriers, offering enhanced solubility, stability, and tumour-targeted delivery *via* the EPR effect.^73^ The study employed a design of experiments (DoE) approach using a Box–Behnken design to optimize the formulation parameters. The micelles were prepared using saponin as the primary surfactant, glycerol as the co-surfactant, and vitamin-E TPGS as the stabilizing agent. Hyaluronic acid, a natural polysaccharide known for its affinity to CD44 receptors, was used to coat the optimized micelles, enabling active targeting of breast cancer cells. The use of HA as a targeting ligand is well-documented for its specific binding to CD44 receptors, which are overexpressed in many cancer cells, promoting receptor-mediated endocytosis and increasing intracellular drug accumulation. HA-coated nanocarriers improve therapeutic efficacy, reduce toxicity, and enhance drug bioavailability.^74^

The key findings of this study are the successful development of HA-CAP-M. Through DoE, the independent variables (concentration of saponin and glycerol, and sonication time) were optimized to obtain the desired particle size, entrapment efficiency (EE), and drug release profiles. The optimized formulation exhibited an average particle size of 17.8 nm, an EE of 89.3%, and a drug release of 67.24% after 4 h. ATR-FTIR confirmed the absence of chemical interactions between CAP and the excipients, indicating formulation stability, while the Tyndall effect confirmed the successful formation of nanomicelles. TEM analysis indicated spherical and uniformly dispersed micelles with HA-coated micelles having a larger size as a result of the HA layer. It is critical to clarify that the increase in hydrodynamic diameter of HA-coated micelles (HA-CAP-M) is primarily due to the hydration shell and surface coating of high molecular weight hyaluronic acid (HA), not an actual shift in core structural size into the microscale domain as measured by DLS. Furthermore, although the IUPAC Recommendations 2011 provide particular classifications separating “nano” from “micro” depending on particle size, such recommendations are precisely meant for polymer-based dispersion systems. In contrast, the micelles developed in this work are based on non-polymeric surfactants and co-surfactants, so they are beyond the direct scope of polymer colloidal nomenclature. Moreover, in line with current usage in nanomedicine literature,^75–77^ formulations displaying nanoscale core architecture and functional behaviour (such as targeted receptor-mediated delivery) are generally referred to as “nanomicelles”, or “nanostructures”, even if their hydrodynamic size slightly exceeds 100 nm due to ligand corona. Thus, the use of the word “nanomicelles” for CAP-M and HA-CAP-M is in line with the accepted scientific standard and adequately captures the intended usage and formulation design. The critical micelle concentration (CMC) of CAP-loaded micelles was markedly decreased from that of the surfactants, suggesting increased thermodynamic stability and efficient micelle formation with lower surfactant concentrations. Drug release studies confirmed a biphasic release profile, including a rapid release phase and a sustained release phase. The Hixson–Crowell model fits the release kinetics best, indicating surface area and particle size changes as governing factors. HA-CAP-M presented a 5-fold increase in flux (0.391 μg cm^−2^ h^−1^) compared to that of CAP (0.075 μg cm^−2^ h^−1^) and a permeability coefficient (0.0521 cm h^−1^) with an increase in permeability compared to free CAP (0.01 cm h^−1^), reflecting improved permeability. The formulation presented high stability during storage and dilution conditions and HA-CAP-M presented higher stability than uncoated micelles. MTT assay data showed dose-dependent cytotoxicity, with the HA-CAP-M resulting in the lowest IC_50_ value (2.964 μg mL^−1^) as a result of CD44 receptor-mediated endocytosis compared with pure drug CAP (5.929 μg mL^−1^). These results clearly show that the ligand-coated carrier improved the cytotoxicity potential by effective targeting of cancer cells. In addition, AO/PI staining showed that MCF-7 cells treated with HA-CAP-M exhibited significant apoptosis, further supporting its potent anticancer activity and CD44-targeting efficiency.

The hydrodynamic diameter of HA-coated micelles (HA-CAP-M) was approximately 205 nm, as determined by dynamic light scattering (DLS). Transmission electron microscopy (TEM) analysis revealed a core size distribution ranging from 180 to 360 nm, primarily due to the hydration shell formed around the high molecular weight (800 kDa) hyaluronic acid, despite the fact that this size may appear larger than typical nanocarriers. The negative zeta potential value (−26.8 mV), low polydispersity index, and consistent results from dilution stability experiments help to further explain this increase in size by hydration effects instead of aggregation or formulation instability. Notably, recent research has demonstrated that hyaluronic acid-coated nanostructures with sizes ranging from 150 to 250 nm can still achieve enhanced tumour accumulation through the enhanced permeability and retention (EPR) effect, while also enabling active targeting through CD44 receptor-mediated uptake.^78,79^ Moreover, the HA-CAP-M formulation showed effective cytotoxic ability and cellular intake, as indicated by the low IC_50_ value (∼2.96 μg mL^−1^) and unambiguous proof of dead cells seen in AO/PI dual staining experiments. These results show that the slightly larger particle size has no negative effect on biological performance. Preliminary optimisation steps include modification of HA concentration and coating kinetics and lowering the average particle size to ∼160–170 nm without compromising target effectiveness or colloidal stability.

Compared to previous studies, the development of HA-CAP-M in this study demonstrates enhanced drug delivery and therapeutic efficacy through active targeting of CD44 receptors. This strategy improves tumour specificity and reduces systemic toxicity.^80^ Our results are consistent with previous studies on cancer cells in the literature. For instance, studies by Liu *et al.* (2017) and Dartora *et al.* (2020) also highlight the use of HA-coated nanocarriers for targeted cancer therapy, showing improved therapeutic outcomes and reduced side effects compared to conventional drug formulations.^81,82^ Additionally, the findings of the current study showing improved drug release profiles, enhanced permeability, and increased cytotoxicity support the growing body of evidence that receptor-mediated drug delivery *via* HA-functionalized carriers offers substantial advantages over non-targeted therapies^83^ These results are consistent with the literature, where HA-coated formulations increase cellular uptake and enhance anticancer activity, demonstrating the potential of HA-based nanocarriers for efficient cancer treatment.

## Conclusion

This study successfully engineered and optimized HA-coated capecitabine-encapsulated nanomicelles as a novel targeted breast cancer therapy platform. The nanomicelles showed enhanced encapsulation efficiency, stability, and release profile with controlled release. The HA coating facilitated active targeting properties through the specific binding of CD44 receptors, thereby improving the therapeutic effects and selectivity of capecitabine. The capacity of the formulation to circumvent the limitations of traditional CAP delivery (*e.g.*, low permeability and systemic toxicity) was demonstrated. The synergic effect of saponin and glycerol for the formation of such a stable micelle structure with a high loading capacity of the drug was the key. Active targeting using HA enabled higher intracellular delivery and decreased off-target effects. The ability of HA-CAP-M to cause apoptosis was apparent, as shown through *in vitro* cytotoxicity and AO/PI staining. The results highlight the feasibility of HA-functionalized nanomicelles as a stable, biocompatible, and effective cancer drug delivery system for breast cancer. Future directions should encompass *in vivo* experiments to confirm the therapeutic efficacy, determine the pharmacokinetics and biodistribution of HA-CAP-M, and scale up the production of the formulation for clinical use, which can serve as an alternative to conventional chemotherapy to improve patient outcomes and quality of life. By integrating nanotechnology and receptor-mediated targeting, this study provides a significant step forward in advancing personalized and precision medicine for cancer therapy.

## Data availability

The data supporting this article have been included as part of the ESI.†

## Author contributions

Sruthi Laakshmi Mugundhan: writing – original draft, review and editing, conceptualization, data curation, and methodology. Mothilal Mohan: writing – review and editing, validation, and supervision.

## Conflicts of interest

There are no conflicts to declare.

## Supplementary Material

RA-015-D5RA01275A-s001
