# Supplementary material for: Hyaluronic acid-coated capecitabine nanostructures for CD44 receptor-mediated targeting in breast cancer therapy
Source: RSC Adv. 2025 Apr 22;15(16):12653–70. doi: 10.1039/d5ra01275a (PMC12012621; doi:10.1039/d5ra01275a)
Supplement: RA-015-D5RA01275A-s001 [file RA-015-D5RA01275A-s001.pdf]

Supplementary Information (SI) for RSC Advances.
This journal is © The Royal Society of Chemistry 2025

HYALURONIC ACID-COATED CAPECITABINE NANOSTRUCTURES FOR CD44 RECEPTOR-MEDIATED TARGETING IN BREAST CANCER THERAPY

Sruthi Laakshmi Mugundhan[a], Mothilal Mohan[a,*]

[a] Department of Pharmaceutics, SRM College of Pharmacy, SRM Institute of Science and Technology, Kattankulathur-603203, Chengalpattu, Tamil Nadu, India

[*]Correspondence to:

Mothilal Mohan, Department of Pharmaceutics, SRM College of Pharmacy, SRM Institute of Science and Technology, Kattankulathur-603203, Chengalpattu, Tamil Nadu, India; E-mail: mothilam@srmist.edu.in; mothipharma78@gmail.com

SUPPLEMENTARY DATA

1. Methods

1.1. Experimental Design

Table S1 represents the levels of independent variables selected for the design.

	Levels		
**Independent variables**	**-1**	**0**	**+1**
**A : Saponin (mMol)**	1	2.5	4
**B : Glycerol (mMol)**	1	2.5	4
**C : Sonication Time (Mins)**	15	30	45

2. Results and Discussion

2.1. Drug - Excipient Compatibility studies

Figure S1 represents the FTIR Spectrum of pure drug Capecitabine.

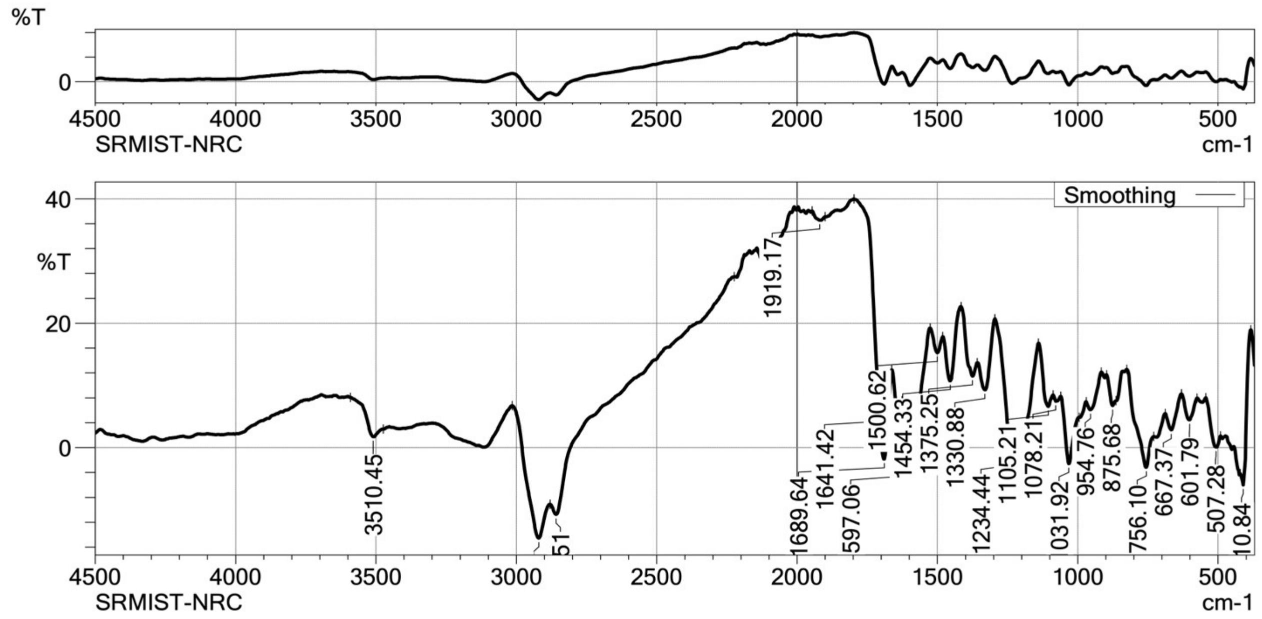

Capecitabine

Table S2 represents the results and interpretation of FTIR spectrum of Capecitabine.

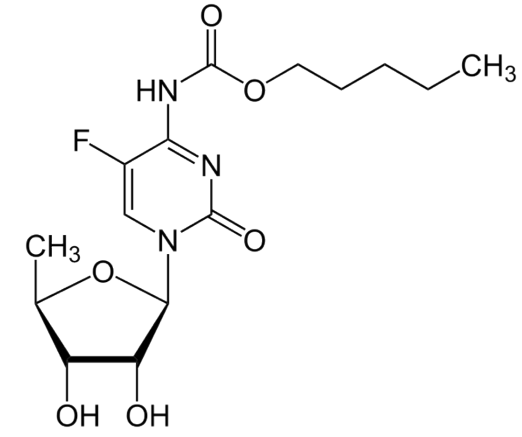

Structure of Capecitabine

WAVELENGTH (cm^{-1})	FUNCTIONAL GROUP	INTERPRETATION
3510.45	Aromatic 1° amine, NH Stretch	Matches the -NH group in Capecitabine's pyrimidine ring

1919.17	Aromatic combination bands	Combination of vibrations from the aromatic rings in Capecitabine’s structure
1689.64	Quinone or conjugated Ketone; Open-chain imino (-C=N-)	Corresponds to the -C=N group in the pyrimidine ring
1641.42	Open-chain imino (-C=N-); Amide	Matches the amide group (-CONH-) and pyrimidine imino group
1454.33	C=C-C Aromatic ring stretch	Matches aromatic stretches in the pyrimidine and phenyl rings
1375.25	Phenol or 3° alcohol	Matches the hydroxyl group (-OH) in the sugar moiety
1330.88	Aromatic 3° amine, CN stretch; Methyne C-H bend (>CH-)	Matches the C-N bonds in Capecitabine's nitrogenous base
1234.44	Aromatic ethers, Aryl - O - Stretch	Matches vibrations of C-O bonds in the ester and sugar moiety
1105.21	Cyclic ethers, large rings, C-O stretch; Aromatic C-H in-plane bend	Matches C-O bonds in the sugar moiety and aromatic C-H bonds
1078.21	Aromatic C-H in-plane bend; Aliphatic Fluoro compounds C-F stretch	Peak corresponds to aromatic C-H bending and C-F stretching
954.76	Aromatic C-H in-plane bend	Matches aromatic C-H bends in the pyrimidine ring and aromatic substituents
756.10	CH 1,3 Disubstitution (meta); Aromatic C-H out of plane bend	Matches meta-substituted aromatic rings in Capecitabine

Figure S2 represents the FTIR Spectrum of physical mixture of Capecitabine and Hyaluronic acid.

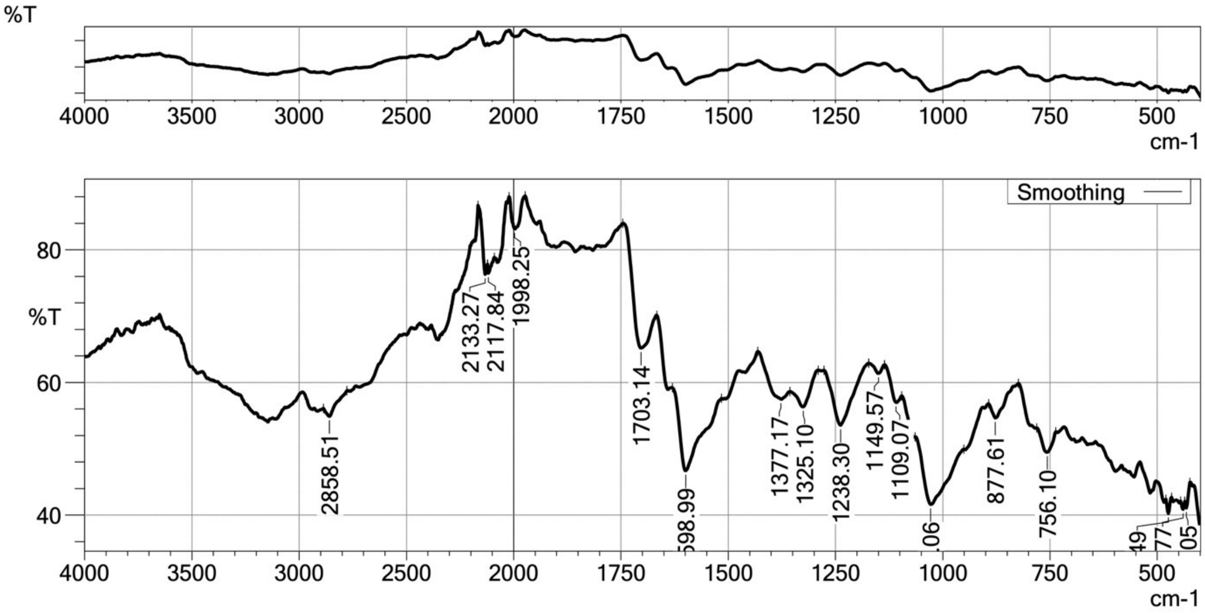

Capecitabine + HA

Figure S3 represents the FTIR Spectrum of physical mixture of Capecitabine and Saponin.

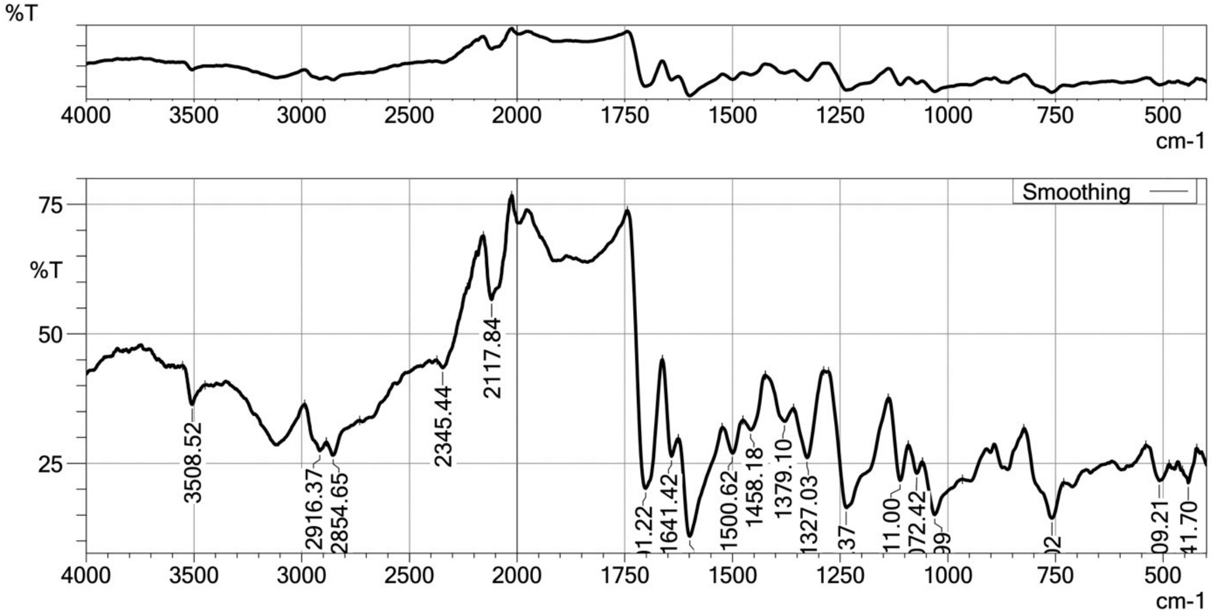

Capecitabine + Saponin

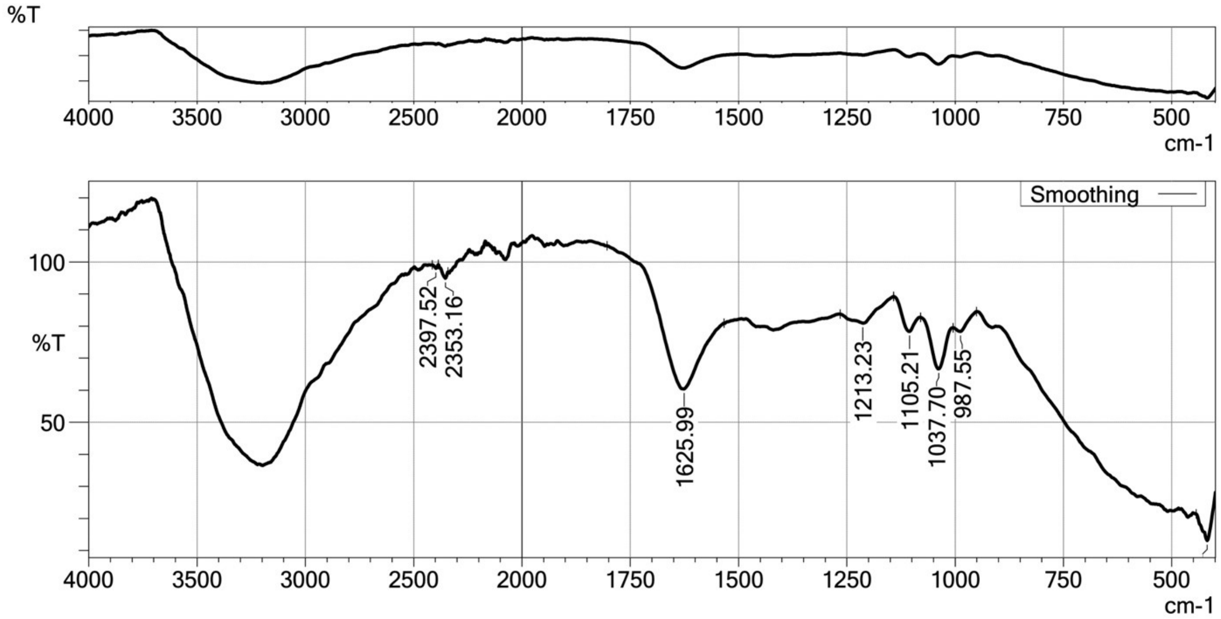

Capecitabine + Glycerol

Figure S4 represents the FTIR Spectrum of physical mixture of Capecitabine and Glycerol.

Figure S5 represents the FTIR Spectrum of physical mixture of Capecitabine and Vit-E TPGS.

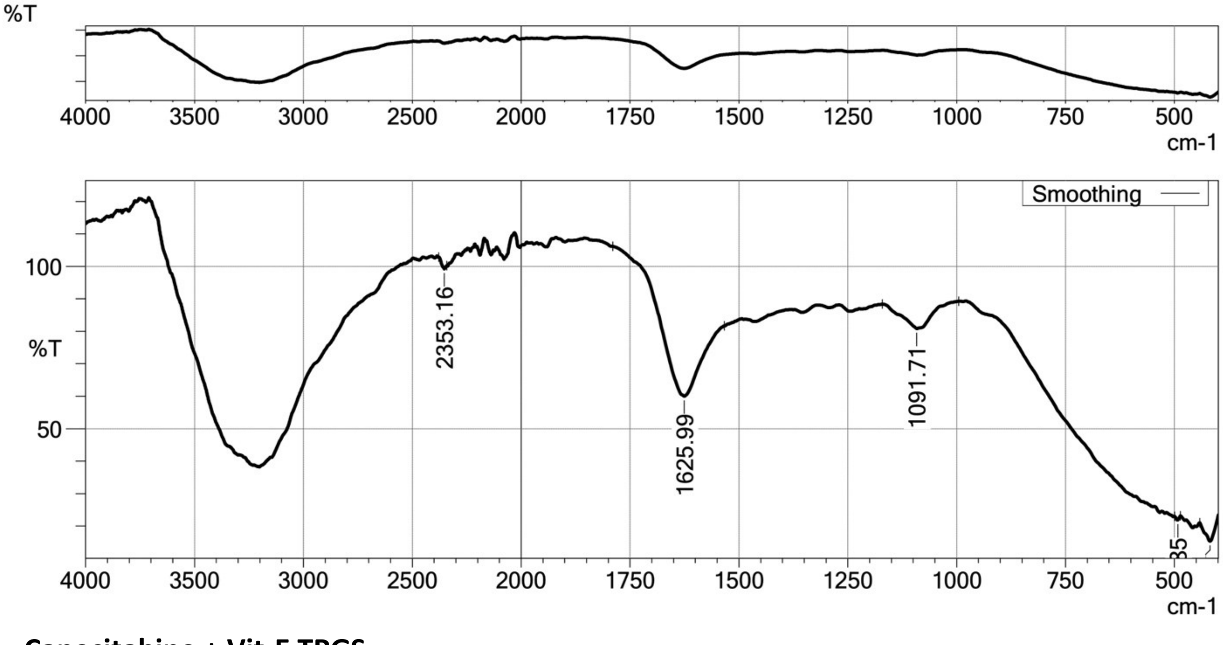

Capecitabine + Vit-E TPGS

Figure S6 represents the FTIR Spectrum of Capecitabine-loaded micelles (CAP-M).

Figure S7 represents the FTIR Spectrum of Hyaluronic acid-coated Capecitabine-loaded micelles (HA-CAP-M).

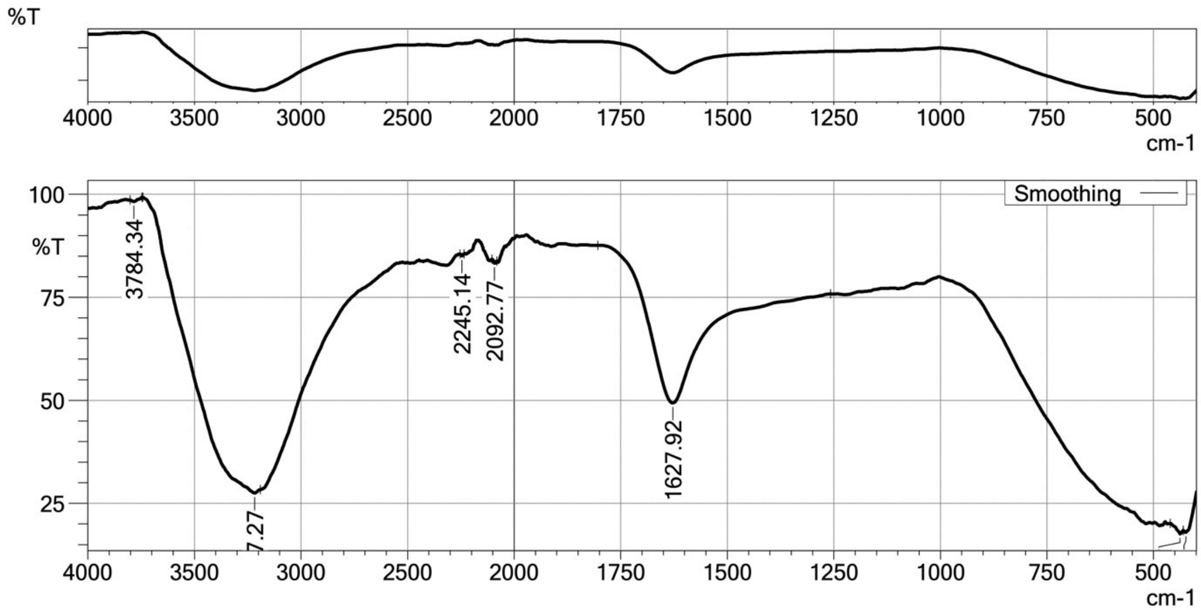

HA-CAP-M

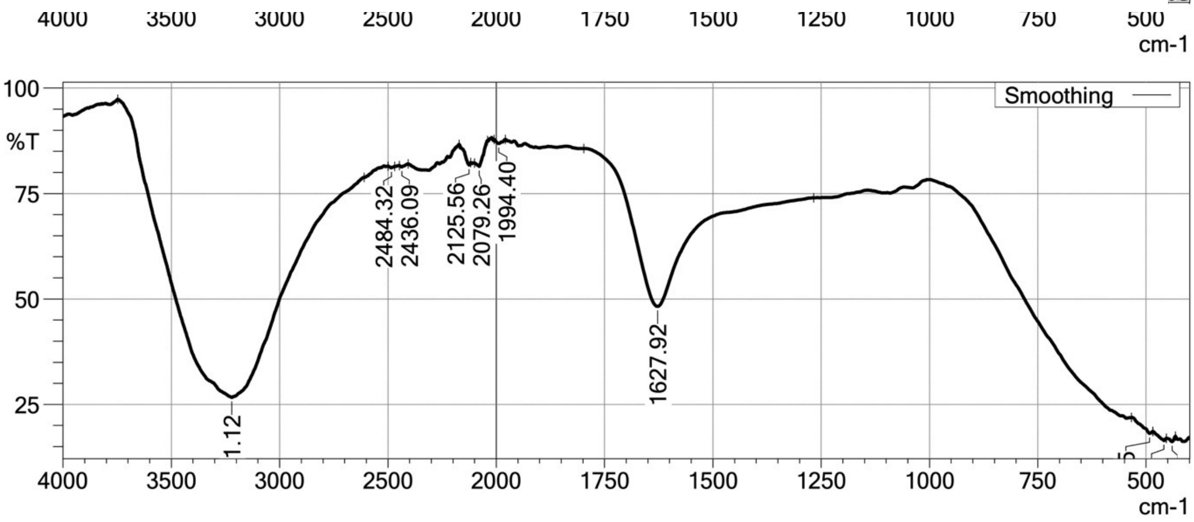

CAP-M

2.2. Optimization

Table S3. Analysis of variance for the quadratic polynomial model for Particle size, EE and *in vitro* drug release after 4 hours

**Source**	**f-Value**	**p-value prob > f**	
**Particle size (nm)**			
**Model**	50.34	0.0002	Significant
A-Saponin	286.60	< 0.0001	
B-Glycerol	14.02	0.0134	
C-Sonication Time	22.00	0.0054	
AB	0.2868	0.6152	
AC	2.10	0.2072	
BC	0.3485	0.5806	
A^2	95.33	0.0002	
B^2	13.26	0.0149	
C^2	33.90	0.0021	
**EE (%)**			
**Model**	906.50	< 0.0001	significant
A-Saponin	7272.78	< 0.0001	
B-Glycerol	85.22	0.0003	
C-Sonication Time	0.5872	0.4781	
AB	10.77	0.0219	
AC	0.0004	0.9846	
BC	0.9642	0.3712	
A^2	355.09	< 0.0001	
B^2	317.28	< 0.0001	
C^2	235.44	< 0.0001	
***In vitro* drug release after 4 hours (%)**			
**Model**	6.41	0.0273	significant
A-Saponin	30.26	0.0027	
B-Glycerol	2.95	0.1463	

C-Sonication Time	0.0664	0.8070
AB	0.5399	0.4955
AC	1.94	0.2224
BC	0.3749	0.5671
A^2	3.97	0.1030
B^2	12.92	0.0156
C^2	7.70	0.0392

Table S4. Predicted models of Capecitabine-loaded micelles

**Models**
**Particle size** = +17.48 - 9.84 A - 2.18 B - 2.73 C - 0.4400 A*B + 1.19 A*C + 0.4850 B*C + 8.35 A^2 + 3.11 B^2 + 4.98 C^2
**Entrapment efficiency =** + 76.22 + 29.63 A + 3.21 B + 0.2662 C - 1.61 A*B + 0.0100 A*C + 0.4825 B*C - 9.64 A^2 -9.11 B^2 - 7.85 C^2
**Drug release after 4 hours** = +66.69 + 3.18 A + 0.9925 B + 0.1487 C - 0.6000 A*B + 1.14 A*C + 0.5000 B*C - 1.69 A^2 -3.06 B^2 - 2.36 C^2

Table S5. Results of regression analysis for responses (Particle size, EE and *in vitro* drug release)

**Response**	**R-squared**	**Adj R- Squared**	**Adeq Precision**	**Lack of fit**
**Particle size**	0.9891	0.9694	20.9185	0.15
**EE (%)**	0.9994	0.9983	81.8498	0.7873
***In vitro* drug release after 4 hours (%)**	0.9203	0.7768	7.4459	0.1032

Table S6. Optimized values obtained from the RSM method and related experimental data at the optimum conditions

Parameter	Predicted by RSM	Experimental Data
**Particle size (nm)**	17.4767	17.8 ± 3.2
**Entrapment Efficiency (EE) (%)**	76.22	76.92 ± 2.41
***In vitro* drug release after 4 hours (%)**	66.6933	66.69 ± 3.43
